# Extraction and identification of new flavonoid compounds in dandelion *Taraxacum mongolicum Hand.-Mazz.* with evaluation of antioxidant activities

**DOI:** 10.1038/s41598-023-28775-x

**Published:** 2023-02-07

**Authors:** Rong Wang, Weihua Li, Cao Fang, Xinxin Zheng, Chao Liu, Qing Huang

**Affiliations:** 1 Institute of Intelligent Machines, Hefei Institute of Intelligent Agriculture, Hefei Institutes of Physical Science, Chinese Academy of Sciences, Hefei, 230031 China; 2 Science Island Branch of Graduate School, University of Science & Technology of China, Hefei, 230026 China; 3 School of Environment and Energy Engineering, Anhui Jianzhu University, Heifei, 230601 China

**Keywords:** Biochemistry, Drug discovery, Molecular biology, Plant sciences

## Abstract

Due to the interest in the potential pharmacological application of dandelion, the chemical constituents and activities of *Taraxacum mongolicum Hand.-Mazz* were studied. Box–Behnken response surface methodology was employed to optimize the protocol for extraction of flavonoid from dandelion. The molecular structures of different flavonoid compounds were acquired and analyzed by liquid chromatography-mass spectrometry (LC–MS) and nuclear magnetic resonance (NMR) spectroscopy. Several major flavonoid compounds were isolated and purified, namely, hesperetin-5′-O-β-rhamnoglucoside, hesperetin-7-glucuronide, kaempferol-3-glucoside, baicalein, hyperseroside, which were extracted for the first time from dandelion. Hesperetin-5′-O-β-rhamnoglucoside was identified as a new type of flavonoid that had never reported in the literature. This new flavonoid has outstanding antioxidant activity, as shown by its IC_50_ value (8.72 mg/L) for scavenging DPPH free radicals. The determination of the structure-related antioxidant activities could be interpreted based on DFT calculations. As such, we have not only illustrated the rich flavonoid contents in *Taraxacum mongolicum Hand.-Mazz*, but also revealed new types of flavonoid compounds in dandelion in terms of structure and antioxidant properties.

## Introduction

Dandelion *Taraxacum mongolicum Hand.-Mazz* (*T. mongolicum Hand.-Mazz*) is a perennial herb which can be used as both medicine and food^1^. It belongs to compositae, alias Huanghuading, or popoding as indicated in the second edition of the Chinese Pharmacopoeia^2^. Nowadays the application of dandelion is increasing, and the research on its chemical composition and pharmacological action is gaining more attention^3^. Generally, dandelion contains a variety of biologically active components, including flavonoids, triterpenes, sesquiterpenes, phenolic acids, sterols and coumarins, with high edible and medicinal value^4^. Particularly, dandelion has antibacterial, anti-inflammatory, antioxidant, hepatoprotective and anti-tumor pharmacological activities^5^. Flavonoid is one class of the main bio-active components in dandelion, which is closely associated with the pharmacological effects of dandelion. For example, the medicinal effects of dandelion such as anti-cancer, anti-aging, liver-protecting, cholagogic and bacteriostasis properties, are either directly or indirectly related to the activities of flavonoids^6^.

**Figure 1:**
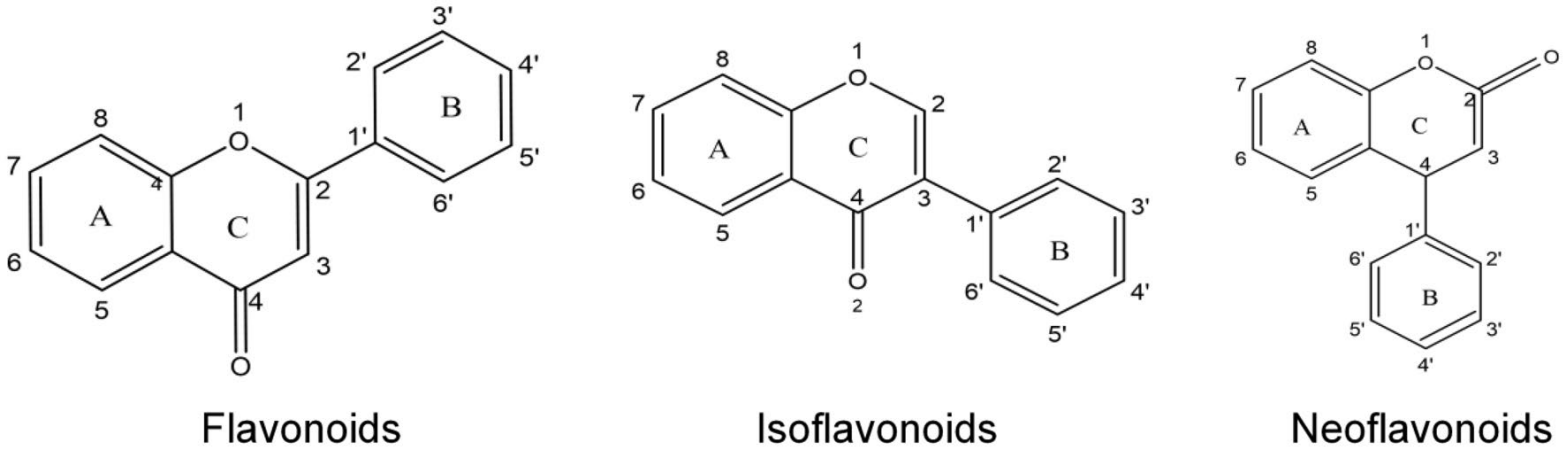
Main parent structures of flavonoids.

In recent years, many studies explored the pharmacological effects and clinical application of dandelion crude preparations, while it is necessary to investigate the pharmacological effects of single chemical compounds such as flavonoids from dandelion. Yanghee et al. conducted experimental studies on the medicinal effect of aqueous extract of dandelion root of *T. mongolicum*^7^. Yuldashev et al. isolated flavonoids including luteolin, quercetin and their derivatives from the roots of medicinal dandelion (*T. officinale Wigg.*)^8^. Shi et al. obtained artemisinin and quercetin from *T. Mongolian* dandelion, and identified two new flavonoids, namely, isoetin-7-O-β-d-glucopyr-anosyl-2′-0-a-l-arabinopyranoside, isoetin-7-0-β-d-gluco^9^. In general, flavonoids stem from secondary metabolic components in plants, which are very important in phytochemistry^10,11^. In terms of chemical structure, there are different flavonoid compounds in which aromatic ring A fuses with pyranone ring C and then connects with another aromatic ring B, and have the basic skeleton characteristics of C6-C3-C6. There are many connecting sites between basic skeleton C ring and B ring, and A ring and B ring often have substituents such as hydroxyl, methoxy, methyl and isopenty, resulting in many different derivatives and active functions^12^. Figure 1 illustrates the flavonoid parent structures, while flavonoids in plants exist mostly in the form of glycosides, that is, hydroxyl groups connected with sugar units and two aromatic rings (A and B). Many studies have proved that the medicinal value of dandelion has a close relationship with the antioxidant activity of dandelion flavonoid^13,14^. However, dandelion flavonoid compounds are complex, and there is a lack of comprehensive analysis of dandelion flavonoids in terms of structure and antioxidant activity. Therefore, it is of great interest to explore the different forms and antioxidant activities of dandelion flavonoid compounds and analyze the corresponding structure–activity relationship.

**Figure 2:**
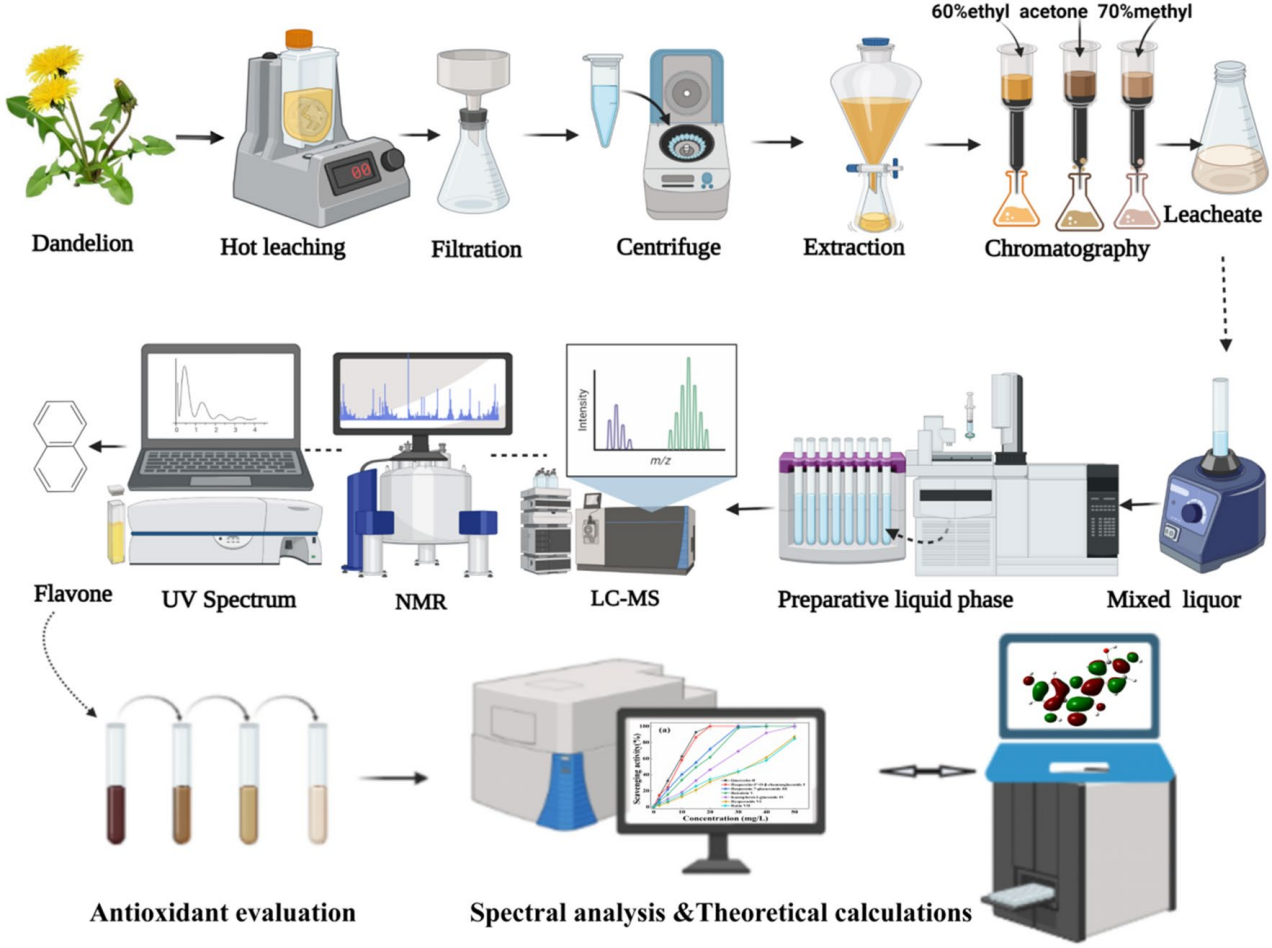
Schematic diagram demonstrating our research on extraction, purification, structural identification and antioxidant evaluation of the flavonoid compounds from *Taraxacum mongolicum Hand.-Mazz*.

In this work, we conducted the study on the extraction and identification of new flavonoid compounds in dandelion *Taraxacum mongolicum Hand.-Mazz.* and evaluated their antioxidant activities. Figure 2 illustrates schematically the experimental procedure. The extraction of the main flavonoid compounds were optimized from *Taraxacum mongolicum Hand.-Mazz* by using response surface methodology. Then, these compounds were purified by preparative high performance liquid chromatography (PHPLC) and analyzed using liquid chromatography-mass spectrometry (LC–MS) and nuclear magnetic resonance (NMR) spectroscopy. The compound structures were determined based on experimental data and also confirmed by theoretical calculations based on density function theory (DFT). The antioxidant activities of the extracted flavonoid compounds were evaluated based on the assessment of the ability of scavenging DPPH free radicals. The structure–activity relationship of the interested flavonoid compounds was therein explored and discussed.

## Results

### Optimization of extraction conditions

**Figure 3:**
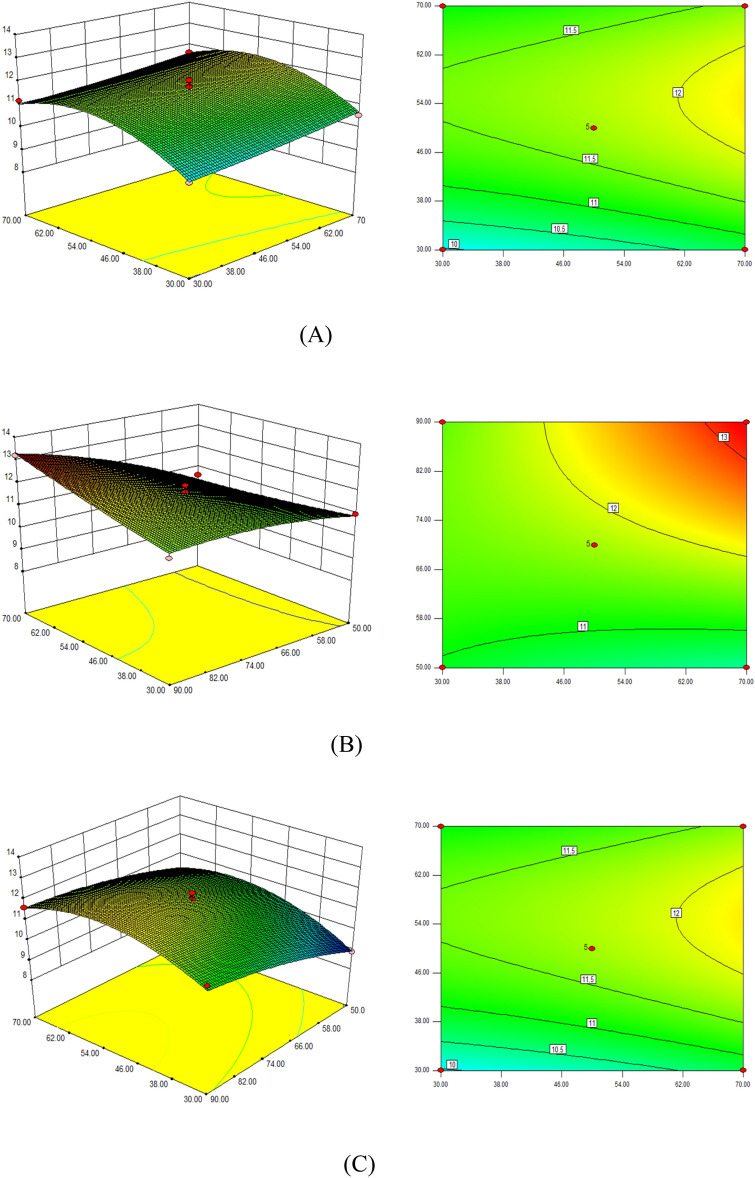
Extraction response surfaces with changes of variable factors: (**A**) Extraction result depending on time and liquid to material ratio, with fixed temperature (80 °C). (**B**) Extraction result depending on time and temperature, with fixed liquid to material ratio (52.56:1 (mL/g)). (**C**) Extraction result depending on liquid to material ratio and temperaturem, with fixed time (70 min).

All experiments were carried out through Design-Expert design response surface optimization experimental scheme. According to the response surface model, the optimum extraction conditions were obtained as follows: extraction time 70 min, liquid-material ratio 52.56:1 (mL/g), extraction temperature 80 °C (the parameters for the response surface optimization are provided in Table S1 and Table S2), and the theoretical yield of total flavonoids of dandelion was 13.31% according to the method reported previously^15^. Considering the simple and practical operation, the optimum extraction process of total flavonoids from dandelion was 70 min, the ratio of liquid to material was 53:1 (mL/g), and the extraction temperature was 80 °C. Each experiment was repeated at least three times under the same conditions. To be noted, the single factor experiments were initially conducted to investigate the effect of related factors on the studied outcome (Fig. S1). As a result, the average yield of total flavonoids of dandelion was 14.12%, and the relative error with the theoretical value (13.31%) was 5.74%, and the prediction ability and feasibility of the model meets the actual expectation (Fig. 3). The quadratic polynomial equation was used for the multiple regression fitting: Y = 11.74 + 0.33A + 0.49B + 0.85C − 0.037AB + 0.59AC − 0.21BC + 0.071A^2^ − 0.98B^2^ − 0.29C^2^.

Tables S1 and S2 list the parameters for the fitting. A, B, C, AC, B^2^ had a significant effect on the total flavonoids yield (P < 0.01), while AB, BC, A^2^, C^2^ had no significant effect on the total flavonoids yield (P > 0.05).

### Identification of new flavonoid compounds

**Figure 4:**
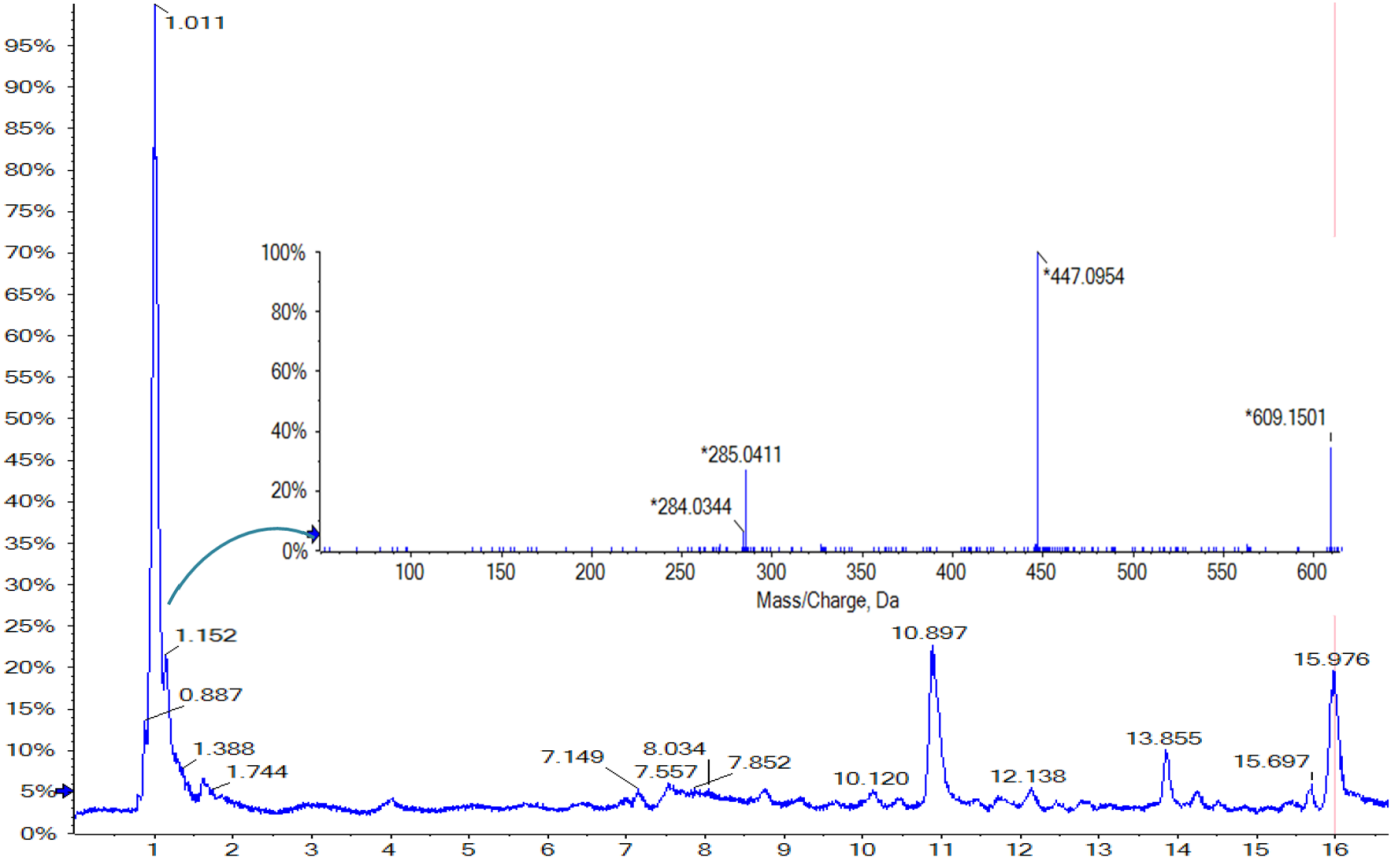
LC–MS peak pattern and corresponding LC-MS^2^ M/Z pattern.

In our study, seven main flavonoid compounds were extracted and purified from dandelion, which were then identified as hesperetin-5′-O-β-rhamnoglucoside (Compound I), quercetin (Compound II), hesperetin-7-glucuronide (Compound III), kaempferol-3-glucoside (Compound IV), baicalein (Compound V), hyperseroside (Compound VI), and rutin (Compund VII) (see details in Figs. S2–S18 for the experimental evidence). Among them, one new type of flavonoid was for the first time identified, namely, hesperetin-5′-O-β-rhamnoglucoside (Compound I), as it had never been reported in the literature. Figure 4 shows the total ion chromatogram and MS spectrum of the new Compound I. The peak time of Compound I is 1.011 min. The liquid chromatography mass spectrum exhibited molecular ion ratio m/z calculated for C_28_H_34_O_15_ (610.56 ([M−H]–: 609.15), m/z 447.1, 285.0 (base peak)). It was determined that the compound was a flavonoid according to its physicochemical properties and ultraviolet spectrum signal.

**Figure 5:**
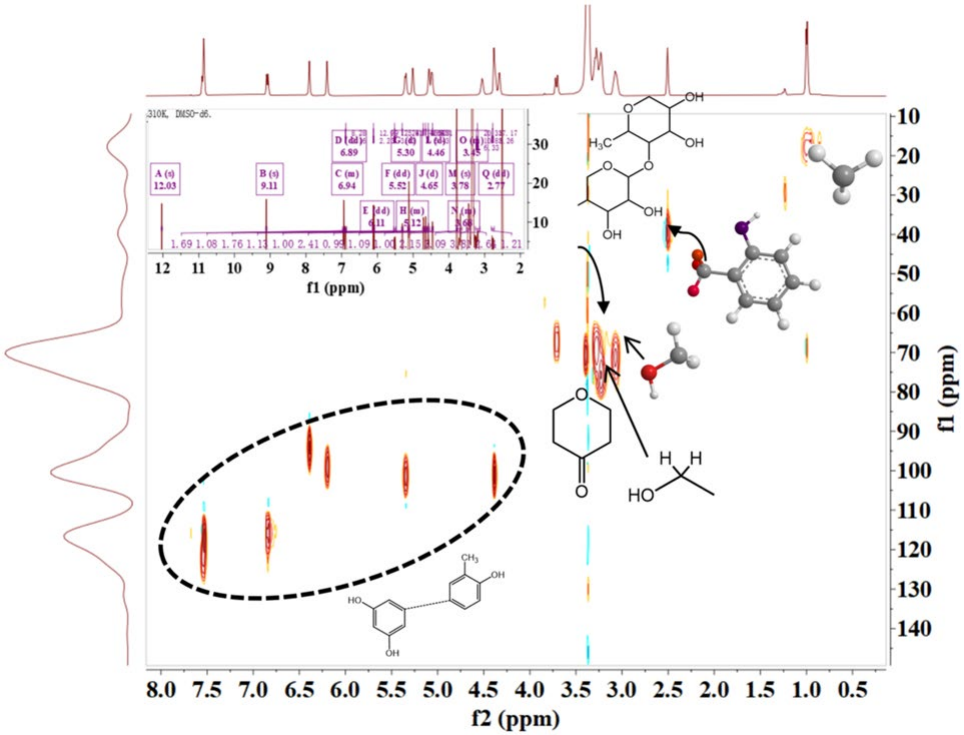
^1^H spectra and ^13^C HSQC NMR spectra of the new Compound I.

The structures of the extracted flavonoid compounds were determined by NMR. Figure 5 shows the 1D hydrogen spectrum and the 2D HSQC spectrum for Compound I. The singlet peaks at 12.03 ppm and 9.11 ppm stand for two protons from phenolic hydroxyl. The multiplet at 6.94–6.11 ppm for five protons form an aromatic ring and yet another multiplet at 5.52–4.46 ppm accounting for thirteen protons from cyclic –CH (glycone ring) were also observed. The singlet peaks at 3.78–3.64 ppm represent the three protons form –OCH_3_ group and a multiplet at 3.66–2.77 for alicyclic hydroxyl group, and a doublet at 2.29 ppm for two protons of –CH_2_OH group attached to glycone ring and the singlet representing three protons at 1.15–1.05 ppm for –CH_3_ were also found. The ^13^C NMR spectrum showed carbon signals at (HSQC, 125 MHz, δ ppm): 145.93, 144.24,131.81, 117.80, 114.07, 111.90, 103.29, 100.35 and 96.22 ppm represented romatic carbon atoms. The peaks at 87.32, 82.42, 78.39, 73.00, 71.75, and 70.11 were assigned for alicyclic carbons. The two carbons from CH_2_OH roup and a methyl group attached to glycone ring were represented at 28.36 and 18.02 ppm respectively. The oxymethine carbon and aliphatic thylene carbons were represented at 74.11 and 32.51 ppm respectively. The methoxy carbon was represented at 49.33 ppm. It was determined as hesperetin-5′-O-β-rhamnoglucoside.

Furthermore, the structure of the new compound was confirmed by using DFT computation with b3lyp/6-311 g (d, p). For determination of the optimal structure of Compound I, ^1^H and ^13^C data were calculated using the DFT/GIAO method through the Gauss 09 program using b3lyp/6-311+g (2d, p) (Table 1). The chemical shift values given by DFT/B3LYP are very close to the experimental data, further verifying the determination of the molecular structure of Compound I.

**Table 1:** Experimental and calculated ^1^H and ^13^C NMR data of new compound.

Position	δH (exp.)	δH (cal.)	δC (exp.)	δC (cal.)
2	4.49	4.39	78.39	78.03
3	6.94	7.28	45.37	43.83
4	–	–		196.44
5	12.03 (s, H, –OH)	12.06	164	166.64
6	6.19 (d, J = 1.9HZ, 1H)	6.14	76.49	97.03
7	9.11(s, 2H)	9.23	160.09	166.32
8	5.3 (d, J = 2.0HZ, 1H)	5.26	96.20	97.04
9	–	–	161.66	165.44
10	–	–	103.29	103.76
1′	–	–	131.81	131.74
2′	6.89 (d, J = 2.148, 1H)	6.75	127.80	124.6
3′	3.85 (s, 3H)	3.73	131.22 (–CH3)	129.86
4′	9.26 (s, –OH)		145.93 (B-cyclic phenol)	145.24
5′	–	–	144.24	144.09
6′	6.11 (dd, J = 8.6, 2.0 Hz)		111.90	–
1″	7.47	7.28	100.35	–
2″	3.31	3.12	71.75	72.90
3″	1.65	1.42	70.11	70.54
4″	–	–	87.32	87.44
5″	1.29 (d, 3H)	1.39	73.00 (–CH3)	72.92
1‴	4.46 (dd, 1H)	4.39	114.07 (C=O)	114.63
2‴	3.54 (dd, 1H)	3.56	76.98 (C–OH)	75.03
3‴	4.6 (d, 1H)	4.56	76.20 (C–OH)	76.54
4‴	4.84 (d, 1H)	4.93	69.50 (C–OH)	67.78
5‴	3.85–4.03 (m, 3H)	3.73–4.1	82.42 (–OCH3)	82.03

**Figure 6:**
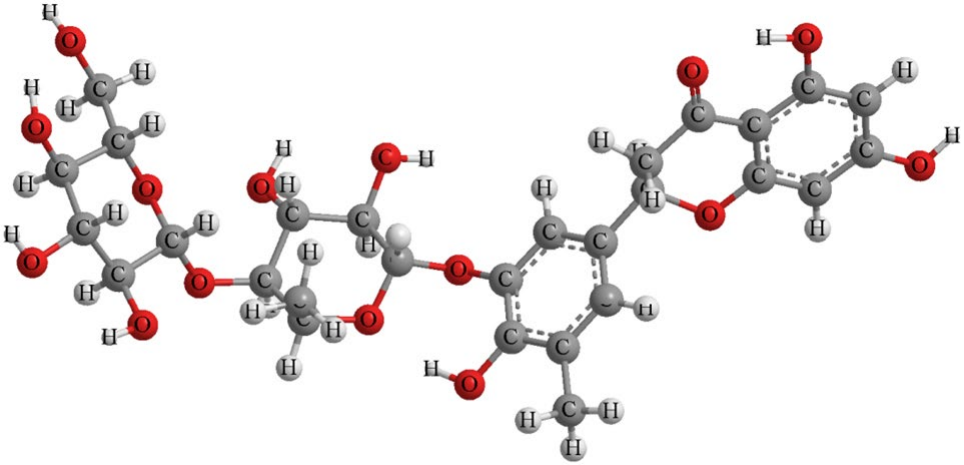
The structure of the new flavonoid compound (Compound I), hesperetin-5′-O-β-rhamnoglucoside.

The new flavonoid compound (I) is a white powdery substance, which can be dissolved in methanol and acetone. It was thus determined as hesperetin-5′-O-β-rham-noglucoside, (2-[3-[3, 4-dihydroxy-6-methyl-5-[3,4,5-trihydroxy-6-(hydroxymethyl) oxan-2-yl] oxyoxan-2-yl]oxy-4-h-ydroxy-5-methylph-enyl]-5,7-dihydroxy-2,3-di-hydrochromen-4-one) (Fig. 6).

### Assessment of antioxidant activity

**Figure 7:**
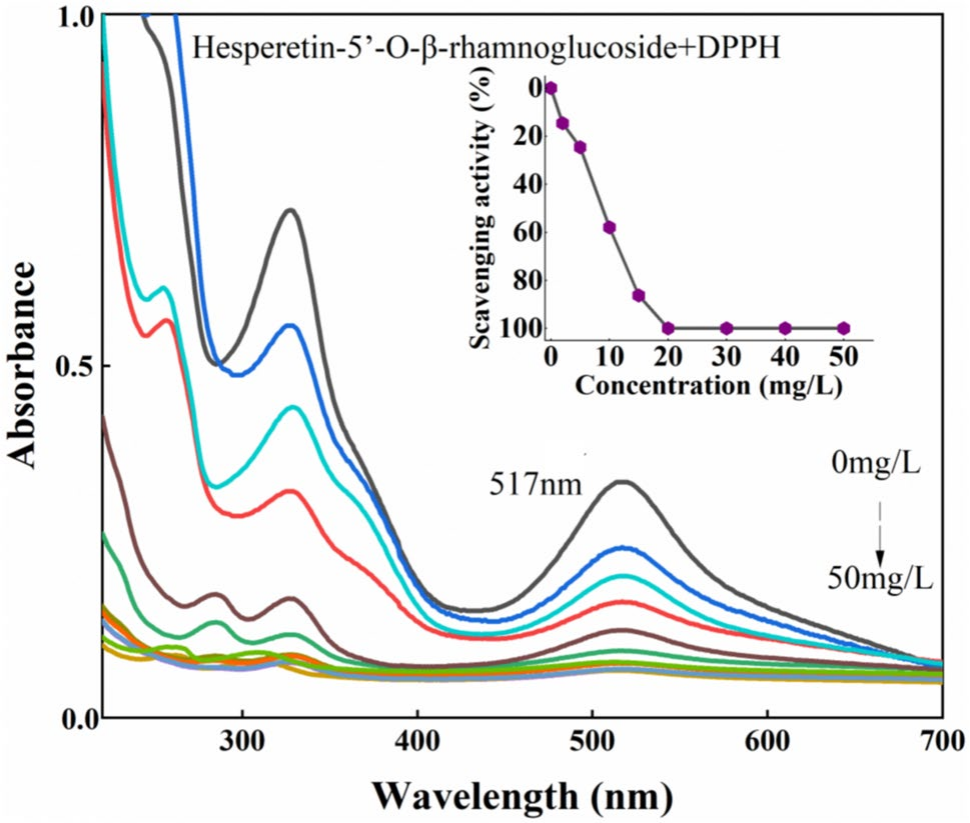
UV–Vis spectra of hesperetin-5′-O-β-rhamnoglucoside-DPPH· complex with oxidant concentration.

The antioxidant activities of the flavonoid compounds were assessed through the DPPH radical scavenging assay. DPPH is stable and easy to handle, which is often used to evaluate the activity of free radicals in scavenging antioxidants^16^. Figure 7 shows the change of absorption in the reaction process of the new component oxidant reactive with DPPH. The absorbance at 517 nm decreased with the increase of the concentration, showing the scavenging efficiency of the new compound.

**Figure 8:**
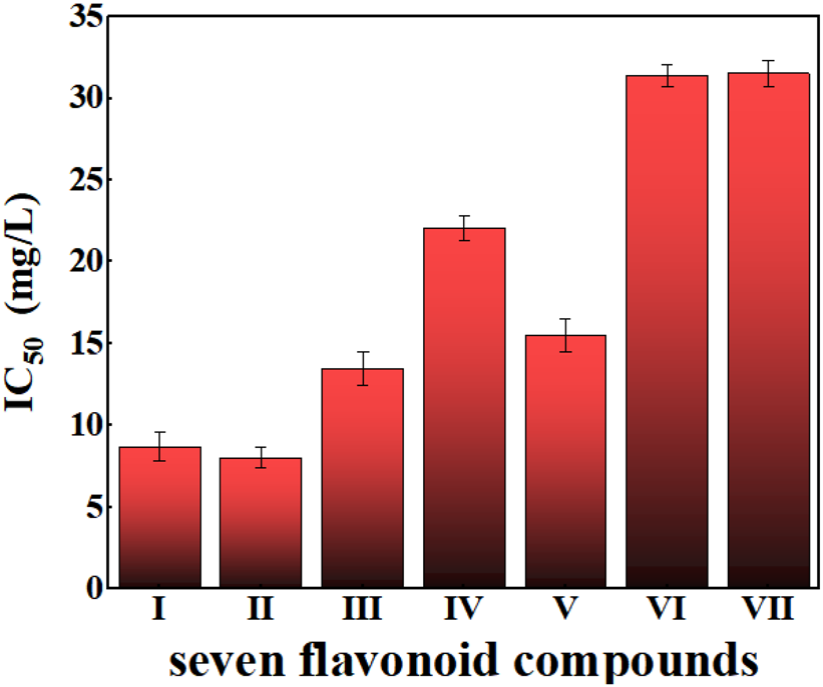
Comparison of the IC_50_ value of the new flavonoid (Compound I) with other extracted flavonoid compounds. All the measurements were made in triplicate, and the IC_50_ values are shown as the mean ± standard deviation in the plot.

For the comparison of antioxidant capacity, IC_50_ was measured which is the concentration of half inhibition rate, that is, the concentration of scavenger when the free radical scavenging rate is 50%. The smaller the IC_50_ value is, the stronger is the scavenging effect or antioxidant capacity^17,18^. Figure 8 shows the results that the IC_50_ of the seven flavonoids in the sequence: quercetin (8.07 ± 0.67 mg/L) < hesperetin-5′-O-β-rhamnoglucoside (8.72 ± 0.88 mg/L) < kaempferol-3-glucuronide (13.49 ± 1.02 mg/L) < baicalein (15.5 ± 0.98 mg/L) < hesperetin-7-glucuronide (22.1 ± 0.76 mg/L) < hysperoside (31.39 ± 0.65 mg/L) < rutin (31.54 ± 0.79 mg/L) (Fig. S19; Table S3).

## Discussion

### Relationship between structure and antioxidant capacity

**Figure 9:**
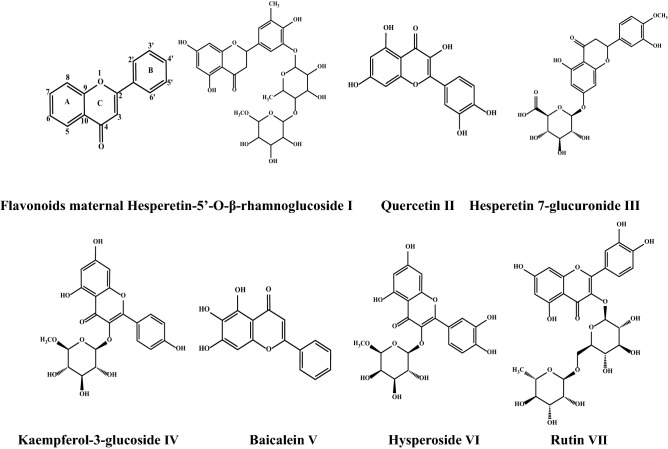
Structures of the extracted flavonoid compounds.

The molecular structures of seven flavonoids are shown in Fig. 9. The activity of phenolic hydroxyl group in ring A is the weakest, while that in ring B is the highest. The ortho-disubstituted group in ring B is the necessary antioxidant group of flavonoids, especially when it is substituted by phenolic hydroxyl group^19^, and the antioxidant activity of 3-OH substitution in C ring is particularly important, among which the glycosylation of 3-OH in C ring is unfavorable, and the stronger the glycosylation degree is, the worse is the antioxidant activity. Therefore, the antioxidant activity is in the order as follows: quercetin > hesperetin-5′-O-β-rhamnoglucoside > hesperetin7-glucuronid, as the steric hindrance of large glycoside group plays a major role in shielding or hindering 3, 4-OH of B ring, resulting in its antioxidant activity reduction^20^. Catherine et al.^21^ also showed that 3-OH of C ring is very important because the hydroxyl group at C-3 position can be isomerized with the double bond at C-2 and 3 position to form a diketone form, producing a highly active CH group, and the unsaturated C ring extends the conjugate system of A ring and B ring, which makes the phenoxy radical more stable and enhances its antioxidant activity. Chun et al.^22^ showed that quercetin and myricetin have strong antioxidant activity due to with this structure.

The experimental results showed that C-ring 3-OH glycosides reduced the antioxidant activity of flavonoids in scavenging DPPH free radicals. The more the number of substituted glycosides, the weaker the antioxidant activity, so the antioxidant activity of monoglycosides substituted flavonoids is better than that of diglycosides. As a result, the antioxidant activity is in the order: kaempferol-3-glycoside > hesperidin > rutin. Furthermore, It was found that the position of phenolic hydroxy group had more influence on the difference of antioxidant activity than the number of phenolic hydroxyl group. The existence of B ring phenolic hydroxyl group greatly improved the antioxidant activity, and the addition of B ring glycoside substituent has a positive effect on antioxidant activity^23,24^, so the antioxidant activity of glycoside substitution of C cyclic hydroxyl group would be unfavorable in the antioxidant capacity. Therefore, the antioxidant activity of baicalein is larger than that of kaempferol-3-glycoside.

Moreover, frontline orbital correlation coefficients can be used to characterize molecular antioxidant activity in quantum chemistry calculations^25,26^. According to molecular orbital theory, frontier orbitals include the highest occupied orbital and the lowest empty orbital, which are closely related to the reactivity of molecules. The highest occupied orbital energy (HOMO) characterizes the electron-giving ability of molecules, that is, the larger the HOMO, the stronger the electron-giving ability of molecules. The lowest empty orbital energy (LOMO) characterizes the ability of molecules to accept electrons, that is, the smaller the LOMO, the stronger the ability of molecules to accept electrons^27,28^. The frontline orbital energy level difference Δ E (Δ E = LUMO–HOMO) characterizes the energy required by molecules from ground state to excited state. The smaller the energy level difference is, the easier is the transition of electrons, so the stronger is the reactivity of molecules^29^. The frontier orbital energy levels of three difference molecules with strongest oxidation resistance were calculated. According to the data in Table 2, the HOMO energy level (− 5.738656 eV) of hesperetin7-glucuronide is higher than that of the other two compounds, and the electrons in this orbital are the most unstable and easy to lose. From the point of view of ΔE = (LUMO–HOMO), the molecular energy range is in the order of ΔE (hesperetin7-glucuronide) > ΔE (hesperetin-5′-O-β-rhamnoglucoside) > ΔE (quercetin). The calculated results are in good agreement with the experimental results (Table S3). The results show that the frontier molecular orbital energy level difference can be used as a reliable theoretical parameter to predict the free radical scavenging activity of flavonoids in the same type of molecules^30^.

**Table 2:** The energy of HOMO, LUMO and ΔE = (LUMO–HOMO) of the four flavonoid compounds(eV).

Energy level	Hesperetin-5′-O-β-rhamnoglucoside	Quercetin	Hesperetin 7-glucuronide
E(LUMO) [eV]	− 2.139008	− 2.133296	− 2.188512
E(HOMO) [eV]	− 5.803936	− 5.738656	− 6.084368
ΔE = (LUMO–HOMO)	3.664928	3.60536	3.895856

In addition, according to the molecular dynamical theory, the breaking of chemical bond needs to absorb energy, so that molecules can move from one potential energy surface to another with higher energy potential energy surface^31,32^.

**Figure 10:**
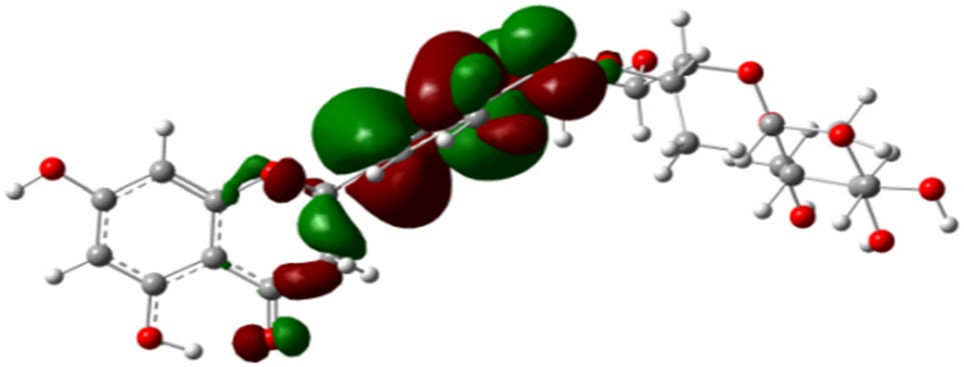
HOMO distribution map of the frontier orbital of the new flavonoid. Red color represents the positive part of the molecular orbital, while the green color represents the negative part.

It can be seen from the molecular orbital (HOMO) (as shown in Fig. 10) that there are many electron clouds in B ring, which is the main chemical reaction site^33^. The most essential physical and chemical parameter is the difference between the heat of formation of free radicals produced by antioxidants and hydrogen extraction reaction OHF (the dissociation energy of hydrogen extraction reaction), that is, the antioxidant activity of flavonoids is to generate phenoxy radicals after hydrogen extraction from parent molecules^34^. Studies have shown that the B ring of flavonoids is the active site of the reaction (Fig. S20), and ortho-substitution can enhance the activity of B ring^35,36^.

**Figure 11:**
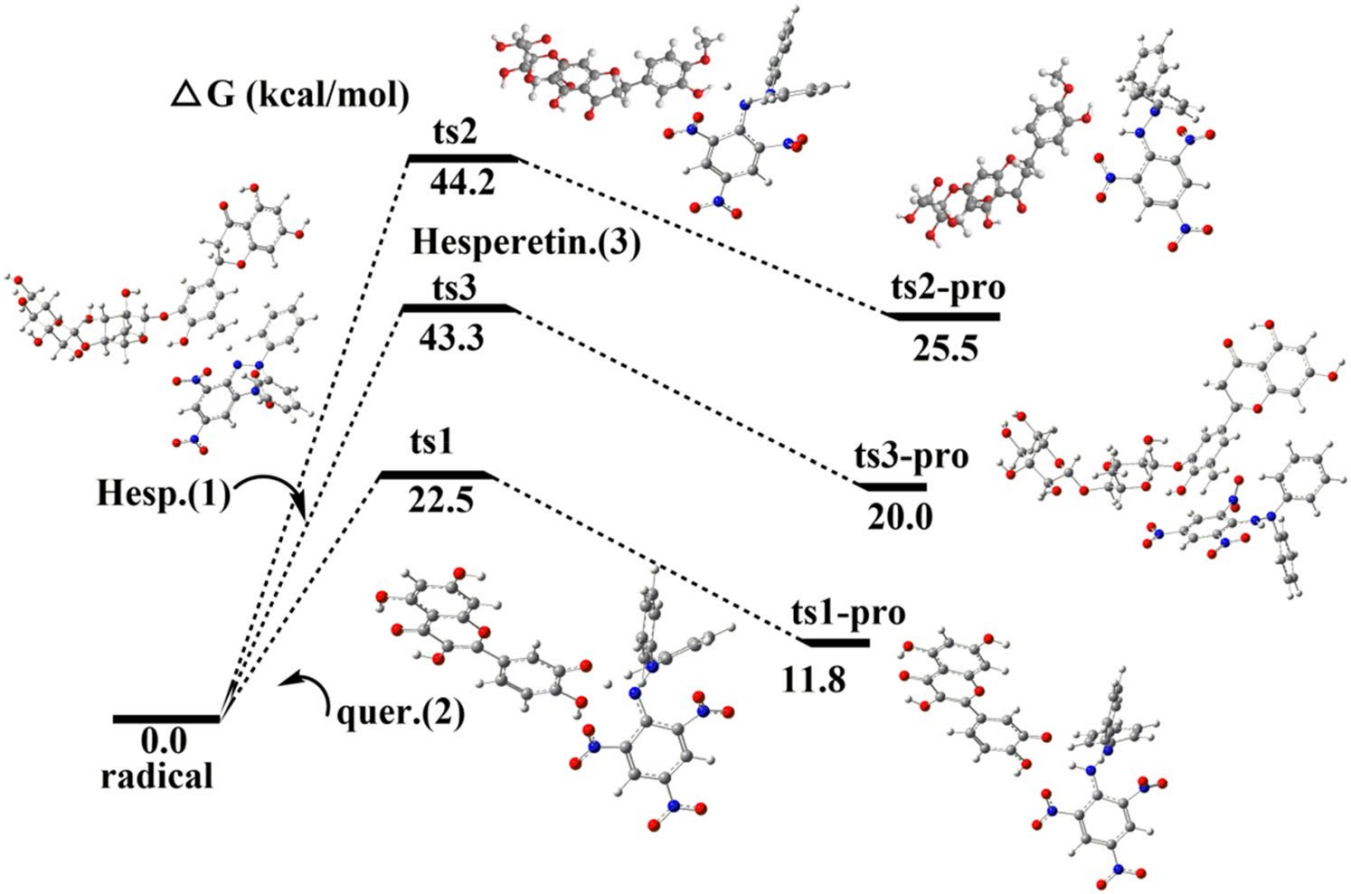
Comparison of dehydrogenation barriers for three flavonoid compounds with 3′ ortho substituents of the ring B.

The three flavonoid compounds have the same substituent at the 4′ position and different 3′ substituents, so the hydrogen-donating energy of the 3′-position functional group was calculated (Fig. 11). Based on the optimized molecular structures, the comparative analysis of enthalpy of hydrogen extraction reaction was also conducted. The calculation results of the hydrogen extraction enthalpy change of the substituent at the 3′ carbon position of the B ring are as follows: quercetin (22.5 kcal/mol) < hesperetin-5′-O-β-rhamnoglucoside (43.1 kcal/mol) < hesperetin-7-glucuronide (44.2 kcal/mol). The results show that the ortho-hydroxyl group of B ring has the strongest activity, mainly because it can carry out the second continuous scavenging reaction of free radicals^37,38^. Therefore, the theoretical prediction results are consistent with the experimental results, showing that DFT method can provide theoretical guidance for the screening of natural flavonoids antioxidants.

## Conclusion

In conclusion, seven major flavonoids were extracted and isolated from the whole herb of dandelion *Taraxacum mongolicum Hand.-Mazz.* by optimized extraction protocol. Hesperetin-5′-O-β-rhamnoglucoside is a new type of flavonoid which was for the first time discovered in this work. The antioxidant activities against DPPH were evaluated and compared. In general, flavonoid has strong activity of scavenging free radicals. The mechanism of scavenging DPPH in vitro is that the substituents on the ring of flavonoids, such as hydroxyl and methoxy, react with free radicals. The molecular orbital energy levels of the flavonoid compounds with stronger antioxidant activity and the hydrogen extraction enthalpy of B ring substituents were reconfirmed by DFT calculation. The theoretical results are consistent with the experimental results, confirming that hesperetin-5′-O-β-rhamnoglucoside has stronger antioxidant activity mainly due to the combined effect of different substituents in B ring, as well as the enhanced hydrogen supply ability. Because these compounds are extracted from natural and edible herbal plant, and they are safe to use as antioxidants in food and cosmetic industries, therefore, this work has not only revealed new flavonoid compounds in dandelion, but also may suggest a new source of potential pharmacological nutrients.

## Methods

### Materials

Dandelion (*Taraxacum mongolicum Hand.-Mazz*) samples were picked from Wulanchabu City, Inner Mongolia. The species was identified by Yuan Ke Yan (inspector of Hohhot Food and Drug Inspection and Testing Center). The experimental material was the whole crushed dandelion including leaf, stem and root. Other chemicals were purchased, which include: rutin standard (HPLC purity ≥ 98% Shanghai McLean Biochemical Technology Co., Ltd.), absolute ethanol (AR Sinopharm), acetic acid (AR Sinopharm), polyamide resin (14–30 mesh Sinopharm), acetone (AR Xilong), methanol (AR Xilong), DMSO-d6 (purity > 99.9%, Shanghai Aladdin Biochemical Technology Co., Ltd.).

### Extraction and isolation

In this experiment, the total flavones of dandelion were extracted by ethanol hot extraction method. The crushed 1 kg dried dandelion was investigated on the basis of ethanol concentration of 60%, extraction time of 30 min, liquid material ratio of 20:1 (mL: g), and extraction temperature of 50 °C. To optimize the dandelion flavonoid extraction, firstly, the single-factor experiments were conducted, wich change of extraction time (30, 40, 50, 60 and 70 min), extraction temperature (40, 50, 60 and 80 °C), and/or the ratio of liquid to material (20, 30, 40, 50 and 60:1 (v/w) mL/g) and ethanol concentration (40%, 50%, 60%, 70%, and 80% (v/w)). One factor was changed while the others were held constant in each experiment. Box–Behnken design (BBD) was employed to determine the best combination of different variables to maximize the flavonoid extraction based on the single-factor experimental results. The proper range for the extraction time (A) and the ratio of liquid to material were selected (B), extraction temperature (C) were determined, and then the response surface methodology was utilized to design the experimental conditions. The independent variables and their levels are given in Table 3. On the basis of the BBD data, a quadratic polynomial model was fitted to correlate the relationship between the independent variables and the response values to predict the optimized conditions.

**Table 3:** Factors adopted in the BBD experiment.

Level	Factor		
	A Extraction (time/min)	B Liquid-to-material ratio (mL/g)	C Extraction (temperature/℃)
− 1	30	30:1	50
0	50	50:1	70
1	70	70:1	80

### Determination of total flavonoids in dandelion

Total flavonoids content was determined following the previously described method of de la Rosa^39^. 1 mL of each sample (250 µg/mL dissolved in methanol) was added with 0.3 mL of sodium nitrite (5%, w/v) and aluminum nitrate (10%,w/v), shaken well, and allowed to stand for 5 min. Then, 4 mL sodium hydroxide (4%, w/v) was added without direct exposure to light and the resulting solution was allowed to stand for 30 min. The sample solution was evaluated using UV–Vis micro plate reader and the absorbance at 510 nm. After measuring the absorbance of the extract with the same method, the mass concentration of total flavonoids was calculated according to the regression equation obtained^39^: Total flavonoids yield (%) = C × V × N/M × 100%, where C is the mass concentration of total flavonoids in dandelion extract, mg/mL; V is the volume of dandelion extract, mL; N is the dilution ratio; M is the mass of dry dandelion powder, mg). Total flavonoids content was presented as catechin equivalents per gram of each sample.

### Isolation and purification of dandelion flavonoids

After being activated with polyamide macroporous resin (bottom) + AB-8 macroporous resin (top), the sample solution was put into a glass separation column for initial separation. 42.7 g of crude extract was divided into three parts on average, eluted with 4.2 L of 70% methanol, acetone and 60% ethanol (volume ratio: 300 mL: 1 g) respectively. Every 500 mL of eluent was collected on Shimadzu LC-16P chromatographic column. YMC Pack ODS-A chromatographic column was used, and SPD-20A was used as the detector. The mobile phase was a binary system, phase A was water containing 0.1% formic acid, and phase B was acetonitrile. In water containing 0.1% formic acid, the gradient elution procedure was applied with the parameters set as: 0–2 min, 20% B; 2–7 min, 60% B; 7–10 min, 75% B; 10–14 min, 35% B; 15–17 min, 20% B.

### Identification of extracted compounds

Different flavonoid compounds were analyzed by AB Triple TOFTM 5600 + LC–MS/MS (SCIEX, ShangHai, China) which was enabled with ultra-fast scanning speed and high mass spectrometry resolution and quantitative sensitivity, ensuring that the system can obtain high accuracy mass spectrometry data and quantitative detection limits. The separation of flavnoid substances in liquid chromatography was performed using C_18_ (100 mm × 4.6 mm, 5 µm particle size) reverse-phase column with the column temperature of 25 ℃. The mobile phase consists of 0.1% formic acid in water (A) and 100% acetonitrile (B) and the flow rate was 0.3 mL/min. The gradient settings for the elution program were as follows: 0–1 min, 95%A; 1–20 min, 95–70% A;20–30 min, 70–10% A; 30–35 min, 10% A; 35–36 min, 10–95%A; 36–40 min, 95%A. The injection volume was 10 µL (concentration unknown). The mass spectrum was obtained over heated electrospray ionization source in negative-ion modes. The key parameters were as follows: the spray voltage was + 3.8 and − 2.8 kV and sheath gas flow rate was 35 arbitrary units (arb unit); auxiliary-gas flow rate was 10 arb unit; capillary temperature was 325 °C with the auxiliary-gas-heater temperature was 350 °C. The scan range was from m/z 100 to 100,000, which covers the flavonoid molecular weight. Data acquisition and processing were carried out with Mass Frontier 7.0 and software Xcalibur 4.1 (Waltham, MA, Thermo Scientific), respectively. For the NMR analysis, the spectra were obtained using Bruker AV-500 spectrometers (500 MHz for 1H NMR and 125 MHz for ^13^C NMR) (Bruker, Switzerland). In addition, ultraviolet–visible (UV–Vis) spectroscopy was also performed on Shimadzu UV-2550 UV–Vis spectrophotometer (200–800 nm) for the compound analysis.

### Assessment of antioxidant capacity

For the antioxidant assay, the compounds were tested against the standard oxidant DPPH. 25 mg DPPH was dissolved in 100 mL of absolute ethanol to obtain a stock solution, and a series of extract sample solutions of different concentrations (0, 2, 5, 10, 15, 20, 30, 40, 50 mg/L) were prepared. For the antioxidant activity measurement, the absorbance value at 517 nm was measured after reaction, and all experiment was repeated for three times. The DPPH radical scavenging capacity was calculated as follows: DPPH· scavenging activity (%) = [1 − (A_s_ − A_b_)/A_d_] × 100%, where A_d_ is the absorbance value of 4 mL 50 mg/L DPPH, A_s_ is the solution absorbance value of 4 mL 50 mg/L DPPH ethanol solution plus 1 mL sample, and A_b_ is the absorbance value of 4 mL absolute ethanol solution plus 1 mL sample solution. The evaluation and comparison of free radical scavenging capacity of antioxidants is based on IC_50_ value, which corresponds to the antioxidant solution concentration when DPPH· radical scavenging rate is 50%^40^^.^

### Theoretical calculations

Calculations were performed to assist the understanding and confirmation of the experimental results including structures, antioxidant activities and spectra. All the calculations were carried out using Gaussian 09 package. The calculations in this work were performed by applying the functionals B3LYP with 6-311G++(d, p) basis set. The calculations gave rise to the energy levels and energy ranges of the highest occupied orbital (HOMO) and the lowest empty orbital (LUMO) of the products with the top three oxidation resistance, as well as the hydrogen extraction enthalpy of B ring substituents^41^. In addition, the NMR 1H and 13C spectra were calculated under the condition of Opt + Freq/GIAO^42^.

### Plants materials

The study complies in accordance with relevant guidelines and regulations.

## Supplementary Information

Supplementary Information.

## Data Availability

All data generated or analysed during the current study available from the corresponding author on reasonable request.
