# Supplementary material for: Extraction and identification of new flavonoid compounds in dandelion Taraxacum mongolicum Hand.-Mazz. with evaluation of antioxidant activities
Source: Sci Rep. 2023 Feb 7;13:2166. doi: 10.1038/s41598-023-28775-x (PMC9905065; doi:10.1038/s41598-023-28775-x)
Supplement: Supplementary file 1 — Supplementary Information. [file 41598_2023_28775_MOESM1_ESM.docx]

**Supporting Information**

**Extraction and Identification of new flavonoid compounds in dandelion *Taraxacum mongolicum Hand.-Mazz.* with evaluation of antioxidant activities**

Rong Wang^a,b,c^,Weihua Li^c^, Cao Fang^a,b^, Xinxin Zheng^a,b^, Chao Liu^a,b^, Qing Huang^a,b,⁎^

^a^ *Institute of Intelligent Machines, Hefei Institute of Intelligent Agriculture, Hefei Institutes of Physical Science, Chinese Academy of Sciences, Hefei 230031, China*

^b^ *Science Island Branch of Graduate School, University of Science & Technology of China, Hefei 230026, China*

*^c^ School of Environment and Energy Engineering, Anhui Jianzhu University, Heifei 230601, China*

**Corresponding author:**

Qing Huang, Hefei Institutes of Physical science, Chinese Academy of Sciences, Hefei 230031, China

Email: [huangq@ipp.ac.cn](mailto:huangq@ipp.ac.cn)

**Optimization of extraction conditions.** Single factor experiment results (Fig. S1)


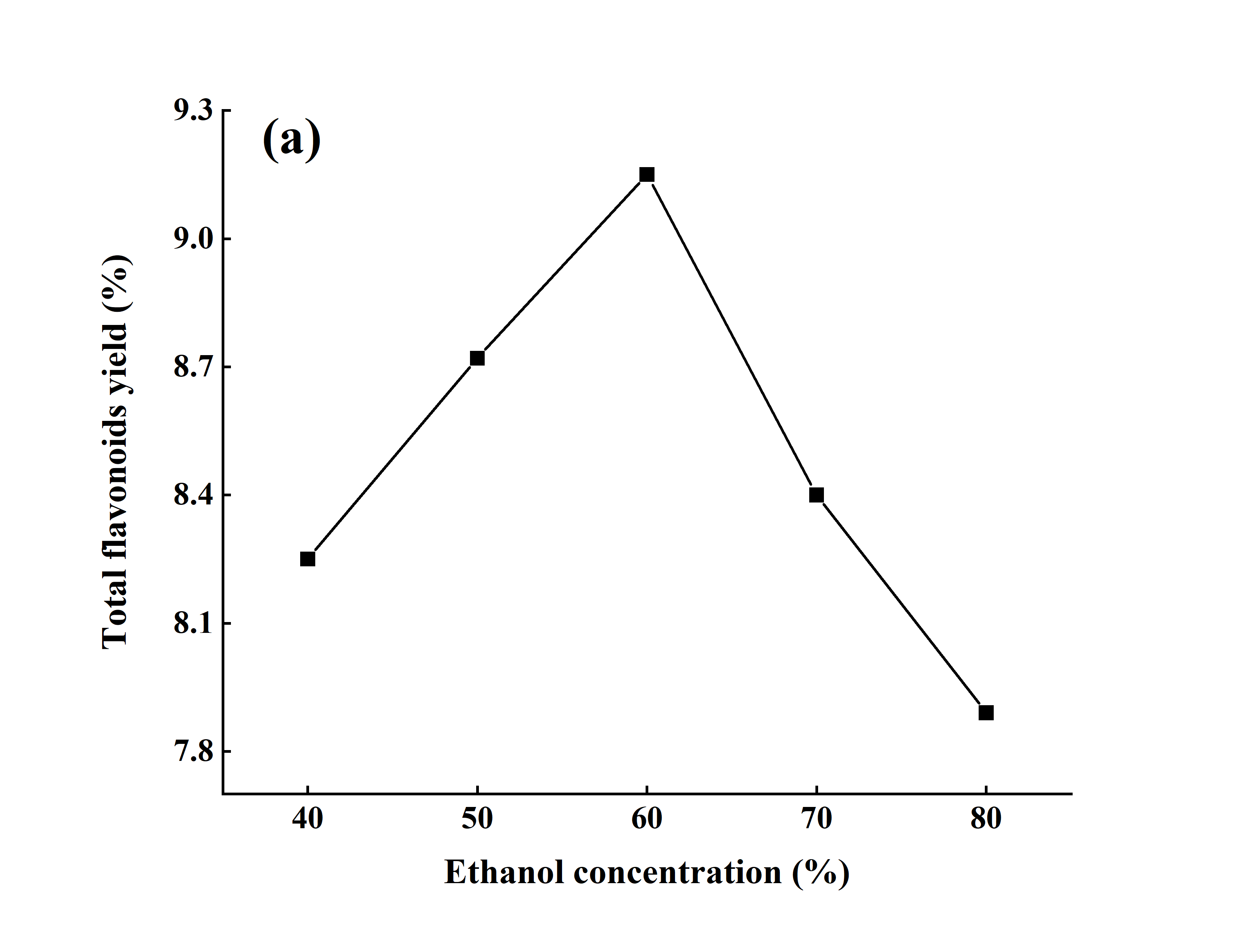

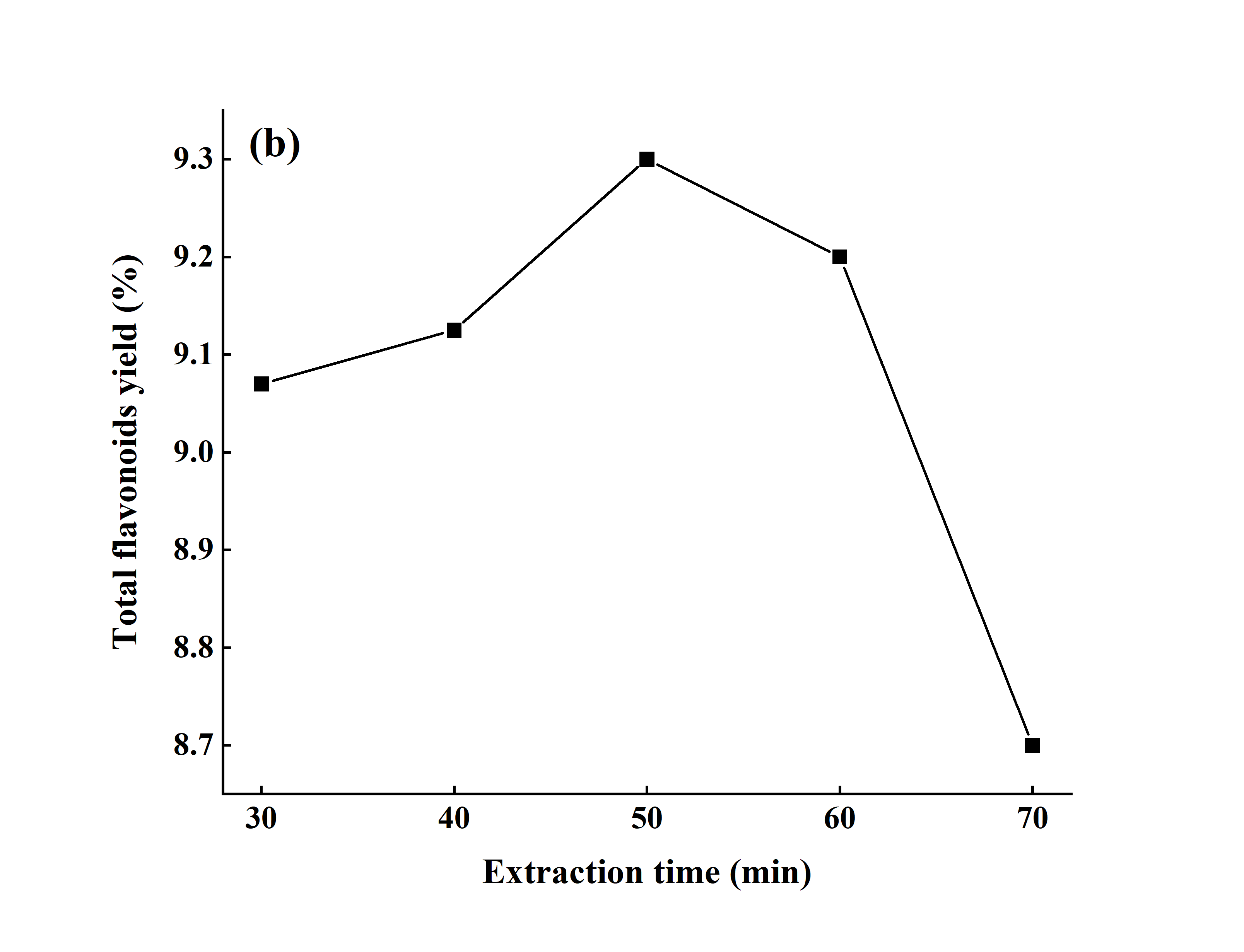


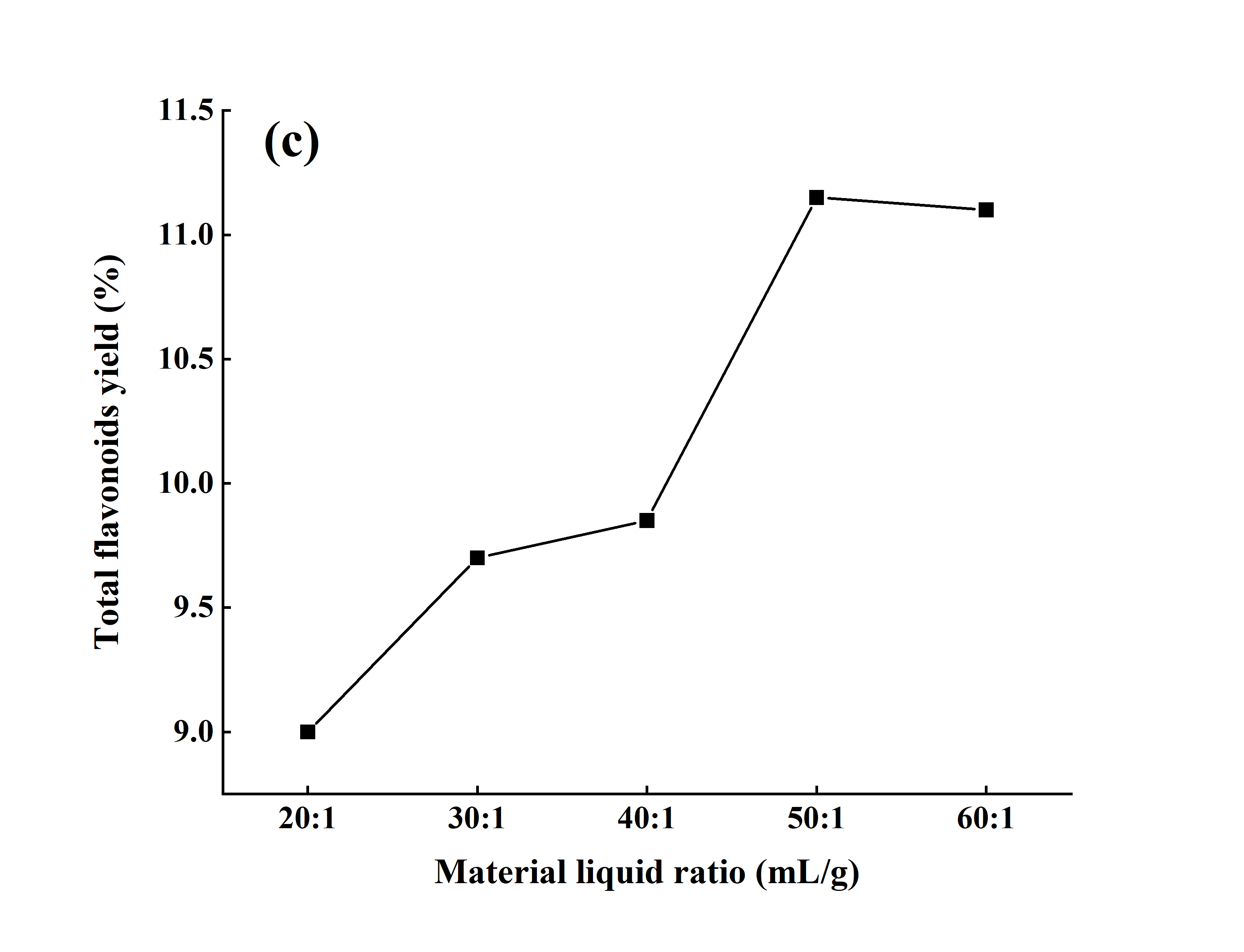

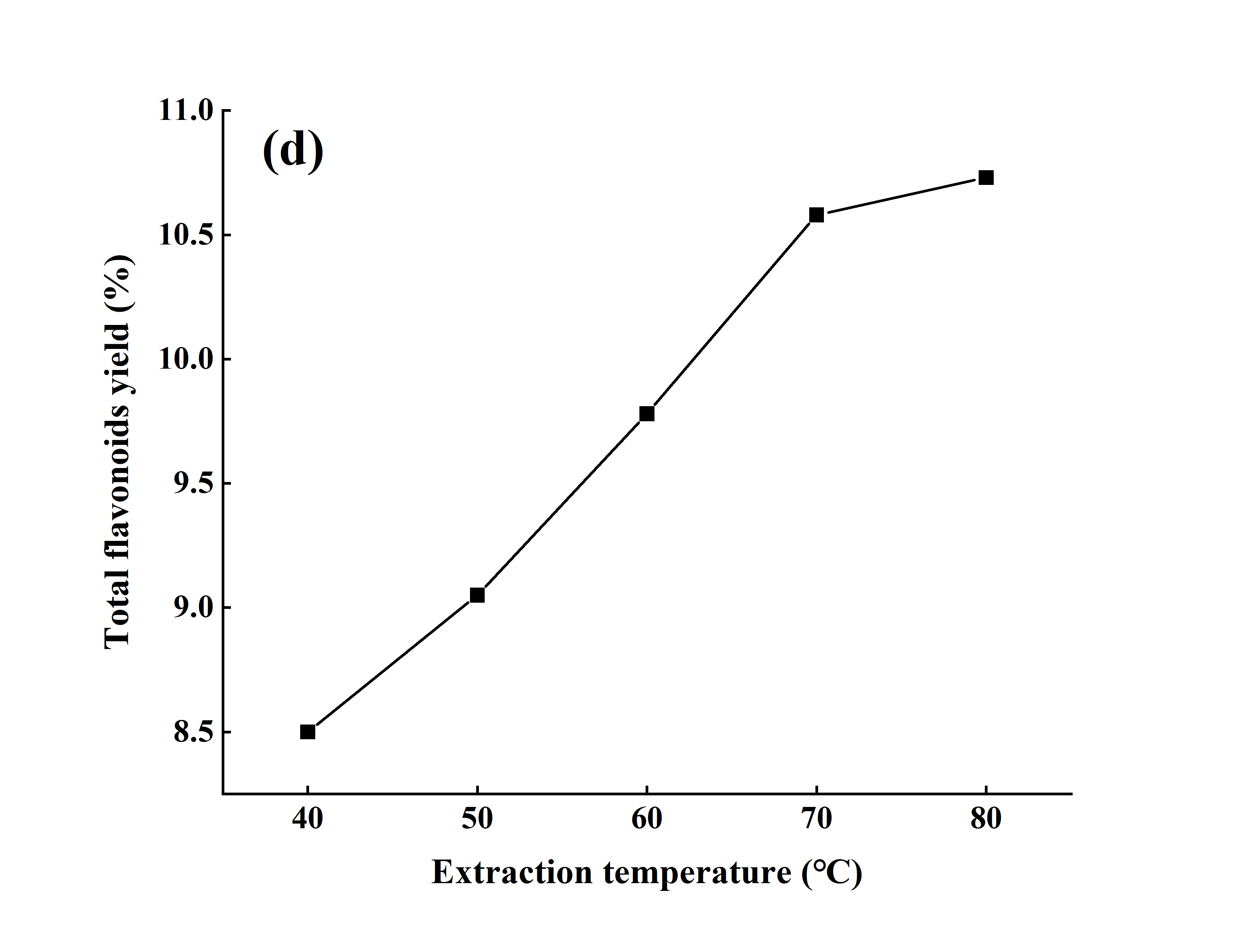


**Figure S1.** Single factor extraction experiment results,(a) Effect of Ethanol Concentration on the Yield of Total Flavonoids from Taraxacum, (b) Effect of Extraction Time on the Yield of Total Flavonoids from Taraxacum, (c) Effect of Liquid to Material Ratio on the Yield of Total Flavonoids from Taraxacum, (d) Effect of Extraction Temperature on the Yield of Total Flavonoids from Taraxacum.

As shown in Fig. S1(a), the yield of total flavonoids of dandelion first increases and then decreases with the ethanol concentration. When the ethanol concentration is 60%, the yield of total flavonoids of dandelion reaches the maximum value of 9.04%, and then decreases with the increase of ethanol concentration. The reason may be that when the ethanol concentration is low, the increase of ethanol concentration is conducive to the dissolution of flavonoids in dandelion cells, but 60% ethanol concentration is a critical point. Later, with the continuous increase of ethanol concentration, some impurities in dandelion cells that are easily soluble in ethanol will also be dissolved one after another, competing with the total flavonoids for solvent, thus reducing the total flavone yield^1^. Therefore, the ethanol concentration is selected as 60%.

As shown in Fig. S1(b), the flavone yield gradually increased from 30 min to the maximum value in 50 min, and then the total flavone yield began to decline with the increase of extraction time. The reason may be that the solvent is not saturated at the beginning, and the total flavonoids are not completely dissolved, leading to the highest yield of total flavonoids. However, with the increase of extraction time, the total flavonoids may be partially decomposed in the continuous heating process. After 60 minutes, the flavone yield accelerated to decline, which may be because the flavones in dandelion are basically completely dissolved. At this time, the flavone dissolution rate is lower than its decomposition rate, thus reducing the total flavone yield significantly^2^. Therefore, the initial extraction time is 50 min.

As shown in Fig. S1(c), the liquid material ratio from 20:1 to 50:1 has always shown an increasing trend in total flavone yield, but after the liquid material ratio from 50:1, the total flavone yield has almost no growth. The reason may be that the more solvents used to extract flavonoids, the more solvents will enter the cells, so that more flavonoids will be dissolved into the solvents^3^. However, after the liquid-solid ratio was 50:1, the mass transfer rate reached saturation due to excessive solvent, resulting in almost no increase in the total flavone yield. Therefore, the liquid material ratio is preliminarily selected as 50:1.

As shown in Fig. S1(d), the total flavone yield increases uniformly from 40 ℃ to 70 ℃, and the growth trend slows down after 70 ℃. The reason may be that the heat movement of macromolecules increases with the increase of temperature, which increases the mass transfer rate^4^. However, too high temperature may also cause the decomposition of total flavonoids. Because the extraction time is too short, the growth trend is only slowed down. If the extraction time is too long, the yield of total flavonoids may decrease. Therefore, the initial extraction temperature is 70 ℃.

The single factor experiment can preliminarily determine that the optimal extraction scheme of total flavonoids from dandelion is ethanol concentration 60%, extraction time 50 min, liquid material ratio 50:1 (mL/g), and extraction temperature 70℃. After that, the extraction time, liquid to material ratio and extraction temperature were selected as three factors to conduct response surface optimization experiments.

**Table S1.** Response Surface Optimization Experiential Results (-1, 0 ,1 represents the value of interaction level between dependent variable regulating variables).

| No. | A Time | B Liquid to material ratio | C Temperature | Y Total Flavonoid yield |
| --- | --- | --- | --- | --- |
|  | (min) | (mL/g) | (℃) | (%) |
| 1 | 0 | 1 | 1 | 11.65 |
| 2 | 1 | -1 | 0 | 10.55 |
| 3 | 0 | 1 | -1 | 10.08 |
| 4 | 0 | 0 | 0 | 11.56 |
| 5 | 0 | -1 | 1 | 11.27 |
| 6 | -1 | 0 | -1 | 11.00 |
| 7 | 0 | 0 | 0 | 11.81 |
| 8 | 1 | 0 | 1 | 13.21 |
| 9 | -1 | 1 | 0 | 11.18 |
| 10 | 1 | 1 | 0 | 11.65 |
| 11 | -1 | -1 | 0 | 9.93 |
| 12 | 0 | 0 | 0 | 11.47 |
| 13 | 0 | -1 | -1 | 8.87 |
| 14 | 0 | 0 | 0 | 11.77 |
| 15 | 1 | 0 | -1 | 10.61 |
| 16 | -1 | 0 | 1 | 11.25 |
| 17 | 0 | 0 | 0 | 12.07 |

For the definition of -1, 0, 1:

| Level | Factor | | |
| --- | --- | --- | --- |
|  | A Extraction (time/min) | B Liquid-to-material ratio (mL/g) | C Extraction (temperature/℃) |
| -1  0  1 | 30  50  70 | 30: 1  50: 1  70: 1 | 50  70  80 |

**Table S2.** Analysis of variance of the second-order regression equation for response surface Design.

| Source of variation | Sum of square | Degrees of Freedom | Mean square | F value | P value | salience |
| --- | --- | --- | --- | --- | --- | --- |
| Model | 14.73 | 9 | 1.64 | 23.97 | 0.0002 | ** |
| A | 0.88 | 1 | 0.88 | 12.96 | 0.0087 | ** |
| B | 1.94 | 1 | 1.94 | 28.43 | 0.0011 | ** |
| C | 5.81 | 1 | 5.81 | 85.19 | < 0.0001 | ** |
| A×B | 0.0056 | 1 | 0.0056 | 0.082 | 0.7824 |  |
| A×C | 1.38 | 1 | 1.38 | 20.23 | 0.0028 | ** |
| B×C | 0.17 | 1 | 0.17 | 2.52 | 0.1562 |  |
| A^2^ | 0.021 | 1 | 0.021 | 0.31 | 0.5957 |  |
| B^2^ | 4.04 | 1 | 4.04 | 59.16 | 0.0001 | ** |
| C^2^ | 0.35 | 1 | 0.35 | 5.16 | 0.0573 |  |
| Residual | 0.48 | 7 | 0.068 |  |  |  |
| Lack of fit | 0.26 | 3 | 0.086 | 1.56 | 0.3298 |  |
| Pure error | 0.22 | 4 | 0.055 |  |  |  |
| All items | 15.20 | 16 |  |  |  |  |

Note: ** indicates extremely signified (P < 0.01)

**Identification of extracted compounds**

**Compound I** is a white powdery substance, which can be dissolved in methanol and acetone. Its molecular formula was confirmed as C_28_H_34_O_15_ (Fig. 9) by ESI-MS (m/z 609.1, 447.09, 285.04). It was determined that the compound was a flavonoid according to its physicochemical properties and ultraviolet spectrum signal. The 1H NMR (500MHz, DMSO-d6, δ ppm) spectrum exhibited signals at 12.03 (s, 1H, -OH phenol), 9.11 (s, 1H, -OH phenol), 6.89 (dd, J=2.148, 1H-2'), 6.11 (dd, J=8.6, 2.0 Hz, H-6'), 6.94 (m, 2H-3), 5.3 (d, J=2. 0Hz, H-8), 3.78 (s, 3H), 5.12-4.03 (m, 13H, -CH ring), 3.65-5.12 (m, 6H, -OH alicyclic), 2.77 (dd, 3H, -CH2OH). The 13C NMR spectrum showed carbon signals at (HSQC, 125MHz, δ ppm): 145.93, 144.24,131.81, 127.80, 114.07, 111.90, 103.29, 100.35 and 96.22 ppm (-CH aromatic), 87.32, 82.42, 78.39, 73.00, 71.75, and 70.11 (-CH Alicyclic). It was determined as Hesperetin-5’-O-β-rhamnoglucoside. (2-[3-[3, 4-dihydroxy-6-methyl-5-[3, 4, 5 - trihydroxy -6-(hydroxymethyl) oxan-2-yl] oxyoxan-2-yl]oxy-4-h-ydroxy-5- methylphenyl]-5,7-dihydroxy-2,3-dihydrochromen-4-one) (Fig. 9; S3; Table 2).

**Compound II** is a pale yellow needle-like crystalline substance, which can be dissolved in methanol and acetone. Its molecular formula was established as C_15_H_10_O_7_ ^5^ by ESI-MS (m/z301, m/z179 and m/z151) (Fig. S4). It was determined that the compound was a flavonoid by According to its physicochemical properties and ultraviolet spectrum signal. The compound's 1H NMR (500MHz, DMSO-d6, δ ppm) spectrum exhibited signals at δ 12.49 (s, 1H, 5-OH), 9.56 (s, 3-OH), 10.18 (s, H, 7-OH), 7.67 (d, J = 2.3 Hz, 1H), 9.33 (s, 4’-OH), 7.54 (dd, J = 8.5, 2.2 Hz, 1H, 2’-H), 6.89 (d, J = 8.5 Hz, 1H), 6.41 (d, J = 2.1 Hz, 1H), 6.19 (d, J = 2.1 Hz, 1H), 6’-H, 6.17 (s, H, 6-H).According to the 2D spectrogram of HSQC at C-H intersect, The 13C NMR spectrum showed carbon signals at (HSQC, 125MHz, δ ppm): 176.1 (C-4), 161.8 (C-5), 98.3 (C-6), 163.1 (C-7), 136.5 (C-3), 145.5 (C-4’), 146.5 (C-5’) (Figure S5-S6; S3). so it was determined to be Quercetin (3, 4-dihydroxyphenyl)-3, 5, 7-trihydroxy-4H-chromen-4-one)^6^.

**Compound III** is a pale yellow powder substance, which can be dissolved in methanol and acetone. Its molecular formula was established as C_22_H_22_O_12_ by ESI-MS (477.13 [M-H]−, m/z 301 and m/z 345.09 MSMS)^7^. (Fig. S7). It was determined that the compound was a flavonoid according to its physicochemical properties and ultraviolet spectrum signal. The compound's 1H NMR (500MHz, DMSO-d6, δ ppm) spectrum exhibited signals at δ12.98 (s, 2H), which were at 7.27 (1H, d, J =1.3 Hz), 6.89 (1H, d, J = 8.7 Hz, 277 H-6') and 6.81 (1H, d, J = 8.2 Hz, H-5').6.43 (1H, s, H-6), 6.13 (1H, d, J = 1.8 Hz, H-8), 5.12 (1H, d, J = 7.7 Hz, H-1''), 4.32 (1H, s, H-2), 4.04 (1H, s, H-3), 3.62-3.28 (2H, m, H-2'', 3'').The 13C NMR spectrum showed carbon signals at (HSQC, 125 MHz, δ ppm): 101.4 (C-10), 145.3(C-3', 4'), 132.1 (C-1'), 119.5 (C-6'), 115.5 (C-2'), 115.3 (C-5'), 102.0 (C-1''), 97.4 (C-6), 96.8 (C-8), 89.8 (C-2), 87.4 (C-3''), 86.3 (C-5''), 81.3 (C-2''), 82.7 (C-4''), 76.7 (C-3). so it was determined to be Hesperetin 7- glucuronide (Fig. S8-S9; S3) ((2S, 3S, 4S, 5R, 6S)-3, 4, 5-trihydroxy-6-[[5-hydroxy-(3-hydroxy-4- methoxyphenyl)-4-oxo-2, 3-dihydrochromen-7-yl]oxy]oxane-2-carboxylic acid)^8^ .

**Compound IV** is a pale yellow needle-like crystalline substance, which can be dissolved in methanol and acetone. Its molecular formula was established as C_21_H_20_O_11_ by ESI-MS (m/z 477.09, m/z 284)^9^ (Fig. S10). It was determined that the compound was a flavonoid by According to its physicochemical properties and ultraviolet spectrum signal. The compound's 1H NMR (500MHz, DMSO-d6, δ ppm) spectrum exhibited signals at δ12.43 (1H, s, 5-OH), 10.73 (1H, s, 7-OH), 9.66 (1H, s, 3-OH), 9.37 (1H, s, 4’-OH), 7.5 (1H, d, J=2.1Hz, H-2’), 7.76 (1H, dd, J=2.1, 8.4Hz, H-6’), 6.94 (1H, d, J=8.4Hz, H-5’), 6.47 (d, J =2.1 Hz, 1H, H-8), 6.19 (d, J =2.1Hz, 1H, H-6), 5.26 (d, J =7.5 Hz, 1H, H-1") 3.41 (m, 1H, H-4"). 3.31(dd, J =9.7, 5.5, 2.4 Hz, 1H, H-5"). 3.73 (dd, J =11.9, 5.5 Hz, 1H, H-6a"). 3.89 (dd, J =11.9, 2.4 Hz, 1H, H-6b"). The 13C NMR spectrum showed carbon signals at (HSQC, 125MHz, δ ppm):179.1 (C-4), 165.9 (C-7), 158.5 (C-9), 163.2 (C-5), 150.6 (C-4’), 148.2 (C-3’), 158.8 (C-2), 134.9 (C-3), 121.5 (C-6’), 121.7 (C-1’), 105.9 (C-3', C-5’), 111.8 (C-2’, C-6'), 103.0 (C-10), 96.7 (C-6), 93.5 (C-8), 104.07 (C-1"), 75.74 (C-2"). 78.05 (C-3"), 71.36 (C-4"), 78.42 (C-5"), 62.63 (C-6"). It was characterized as kaempferol-3-glucoside (Fig. S11-S12; S3) (5, 7-dihydroxy-2-(4-hydroxyphenyl)- 3-[(2s,3R, 4s, 5s, 6R)-3, 4, 5-trihydr-oxy-6-(hydroxymethyl)Oxan-2-yl] Oxychro-men-4-one)^10^ .

**Compound V** is a pale yellow needle-like crystalline substance, which can be dissolved in methanol and acetone. Its molecular formula was established as C_21_H_20_O_11_ by ESI-MS (m/z 269.05, 251)^11^ (Fig. S13). The compound's 1H NMR (500MHz, DMSO-d6, δ ppm) spectrum exhibited signals at 12.72 (s, 1H, -OH), 8.90 (s, 1H, OH), 8.09-7.61 (m, 5H, -CH aromatic), 6.95 (s, 1H, -CH aromatic), 6.67 (s, 1H, -CH aromatic). The 13C NMR spectrum showed carbon signals at (HSQC, 125MHz, δ ppm): 182.08 (>C=O), 162.83 (C=C), 153.58, 149.78, 146.90, 131.76, 130.88, 129.28, 129.04, 126.22, 104.40, 104.24, 93.98 (-CH aromatic). It was characterized as baicalein (Fig. S14-S15; S3) (5, 6, 7-trihydroxy-2-phenyl-4H-chromen-4-one)^12^.

**Compound VI** is a Light yellow and light green crystalline powder substance, which can be dissolved in methanol and acetone. Its molecular formula was established as C_21_H_20_O_12_ by ESI-MS (m/z 463.1,301.03,271.02)^13,14^ (Fig.. S16). It was determined that the compound was a flavonoid by According to its physicochemical properties and ultraviolet spectrum signal. The compound's 1H NMR (500MHz, DMSO-d6, δ ppm) spectrum exhibited signals at δ12.63 (s, 1H, 5-OH), 10.87 (s, 1H, 7-OH), 9.72 (s, 1H, 4’-OH), 9.18 (s, 1H, 3’-OH), 7.67 (dd, J = 8.5, 2.2 Hz, 1H, 6'), 7.53 (d, J = 2.3 Hz, 1H, 2'), 6.82 (d, J = 8.4 Hz, 1H, 5'), 6.41 (d, J = 2.1 Hz, 1H, 8), 6.21 (d, J = 2.0 Hz, 1H, 6), 5.38 (d, J = 7.7 Hz, 1H, 1''). The 13C NMR spectrum showed carbon signals at (HSQC, 125MHz, δ ppm): δ 158.5 (C-2), 135.6 (C-3), 179.4 (C-4), 163.1 (C-5), 100.1 (C-6), 166.1 (C-7), 94.8 (C-8), 145.8(C-3’), 149.9(C-4’), 71.2, 73.2, 67.9, 75.8, 60.1 (Glu.), It was characterized as hyperoside (Fig. S17-S18; S3) ((3, 4-dihydroxyphenyl)-5, 7-dihydroxy-3-[(2S, 3R, 4S, 5R, 6R)-3, 4, 5-trihydroxy-6-(hydroxymethyl)oxan-2-yl]oxychromen-4-one)^15^.

**Compound VII** is yellow for crystallizationa substance, which can be dissolved in methanol and acetone. Its molecular formula was established as C27H30O16 by ESI-MS (m/z 609,285 ,301)^16^ (Fig. S19). The compound's 1H NMR (500MHz, DMSO-d6, δ ppm) spectrum exhibited signals at δ12.60 (s, 1H, 5-OH), 10.84 (s, 1H, 7-OH), 9.66 (s, 1H, 4’-OH), 9.17 (s, 1H, 3’-OH), 7.64-7.50 (m, 2H, 2', 6'), 6.85 (d, J = 8.0 Hz, 1H, 5'), 6.39 (s, 1H, 8), 6.20 (d, J = 2.4 Hz, 1H, 6), 5.35 (d, J = 6.2 Hz, 1H, 1''), 5.27 (s, 1H), 5.10 (s, 1H), 5.06 (d, J = 5.9 Hz, 1H), 4.52 (d, J = 5.1 Hz, 1H), 4.41-4.30 (m, 3H), 3.71 (d, J = 11.0 Hz, 1H), 3.32 -3.17 (m, 5H), 3.14-3.03 (m, 2H), 1.00 (d, J = 6.2 Hz, 3H, 6''').The 13C NMR spectrum showed carbon signals at (HSQC, 125MHz, δ ppm): 156.4(C-2), 133.2(C-3), 177.2(C-4), 161.1(C-5), 98.8(C-6), 164.7(C-7), 93.6(C-8), 156.4(C-9), 103.6(C-10), 121(C-1’), 115.2(C-2’), 144.8(C-3’), 148.6(C-4’), 116.1(C-5’), 121.5(C-6’), 101.3、74、76.4、69.9、75.9、66.91(Glc), 100.7、70.5、70.3、71.8、68.2 (Rha). It was characterized as Rutin (Fig. S20-S21; S3) (3, 4-dihydro-xyphenyl)-5, 7-dihydroxy-3-[(2S, 3R, 4S, 5S, 6R)-3, 4, 5-trihydroxy-6-[[(2R, 3R, 4R, 5R, 6S)-3, 4, 5-trihydroxy-6-methyloxan-2-yl]oxymethyl]oxan-2-yl]oxychromen-4-one(4)^17^.


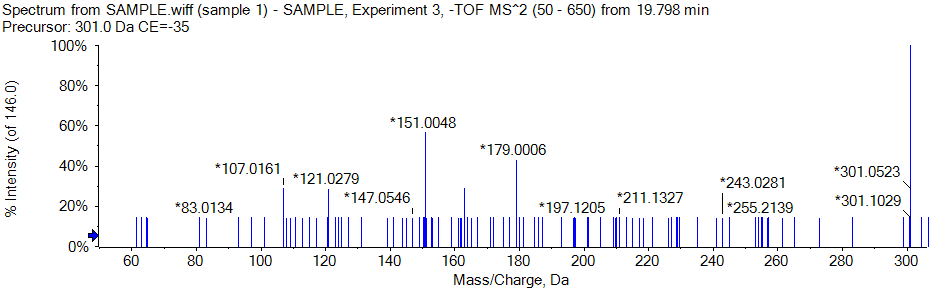


**Figure S2.** The GC-MS spectrum of the compound II.


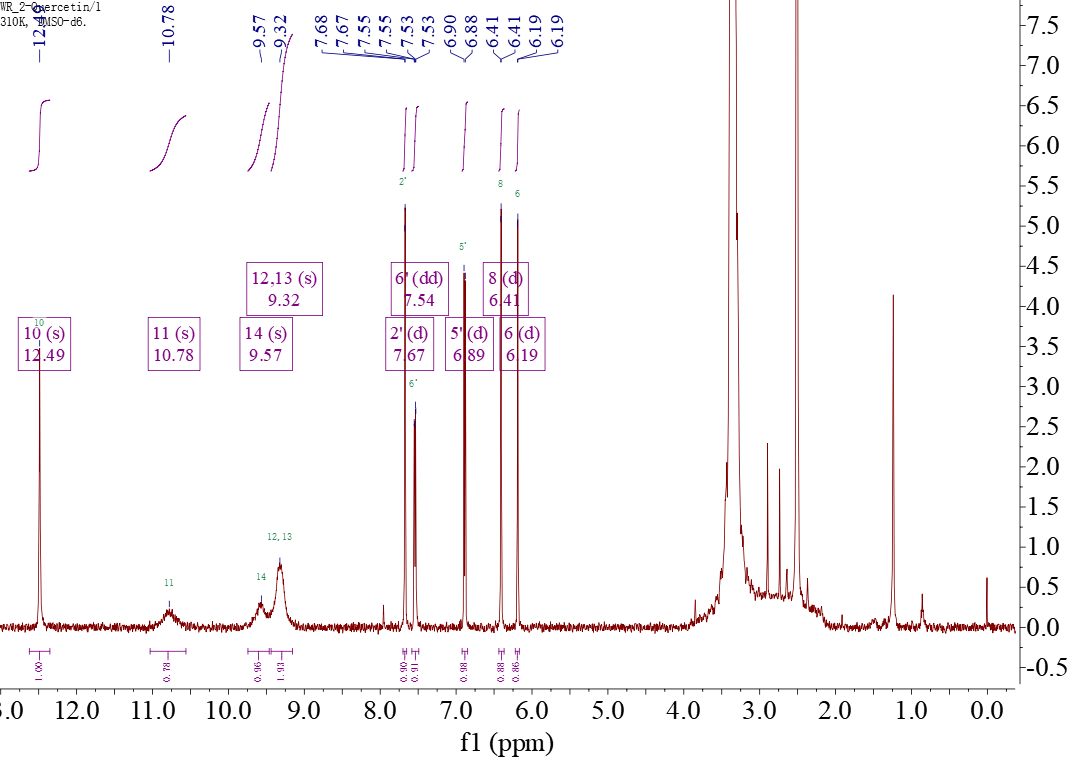


**Figure S3.** The ^1^H NMR spectrum (500 MHz, DMSO-d_6_) of Compound II.


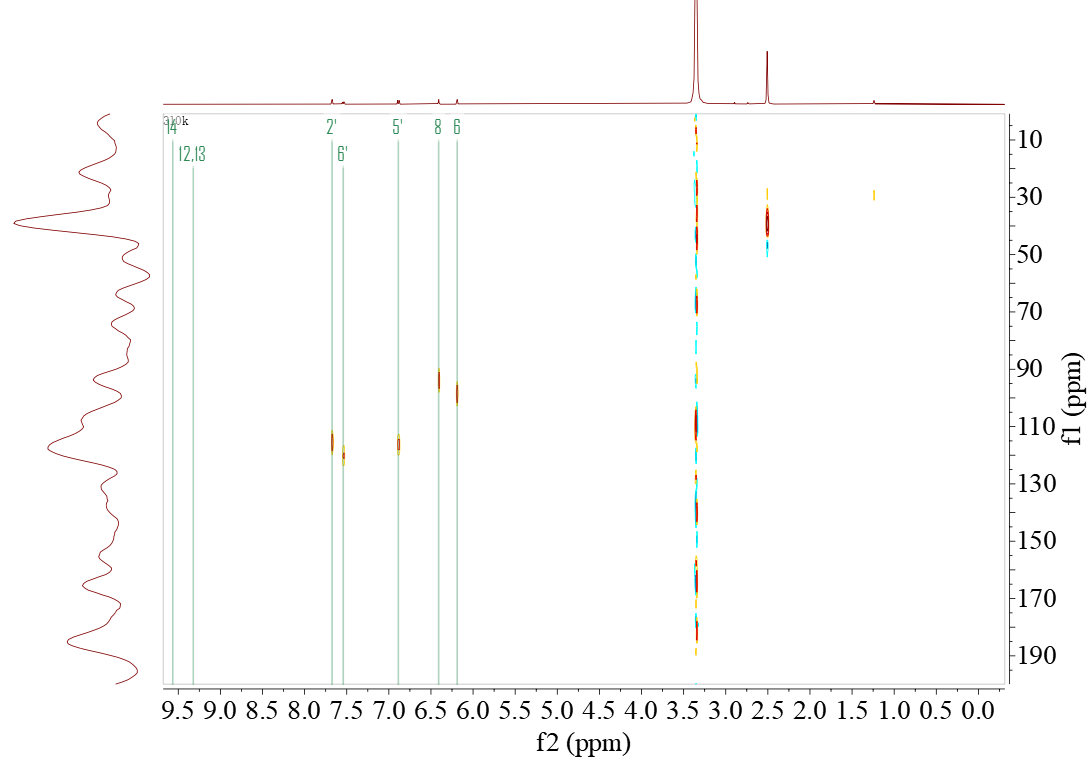


**Figure S4.** The HSQC (^13^C,125 MHz, DMSO-d_6_ ) spectrum of Compound II.


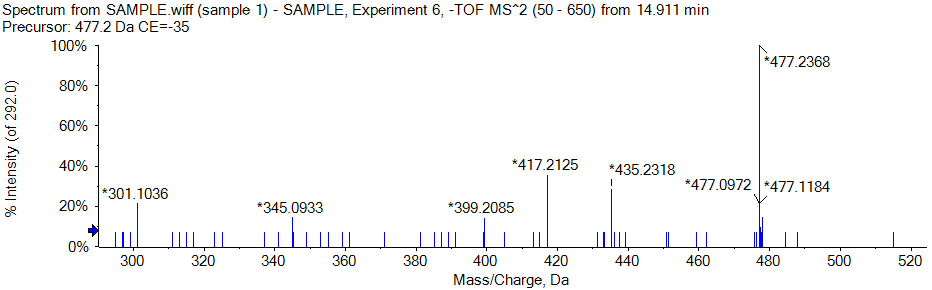


**Figure S5.** The GC-MS spectrum of Compound III.


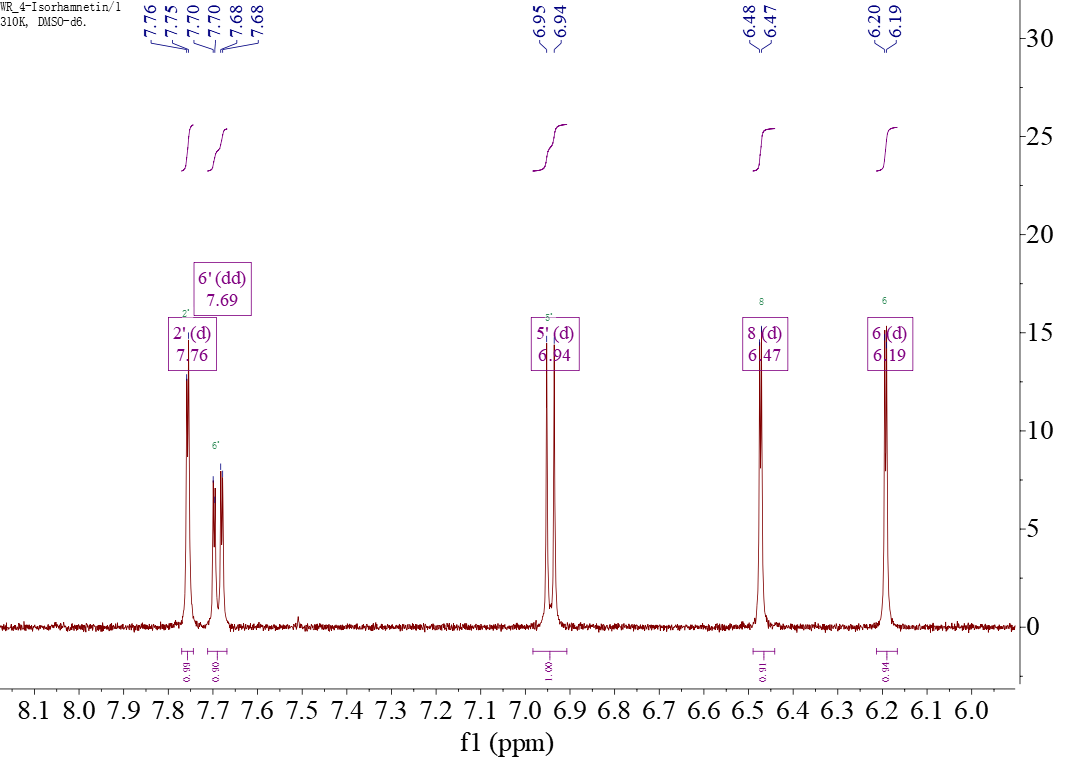


**Figure S6.** The ^1^H NMR spectrum (500 MHz, DMSO-d_6_) of Compound III.


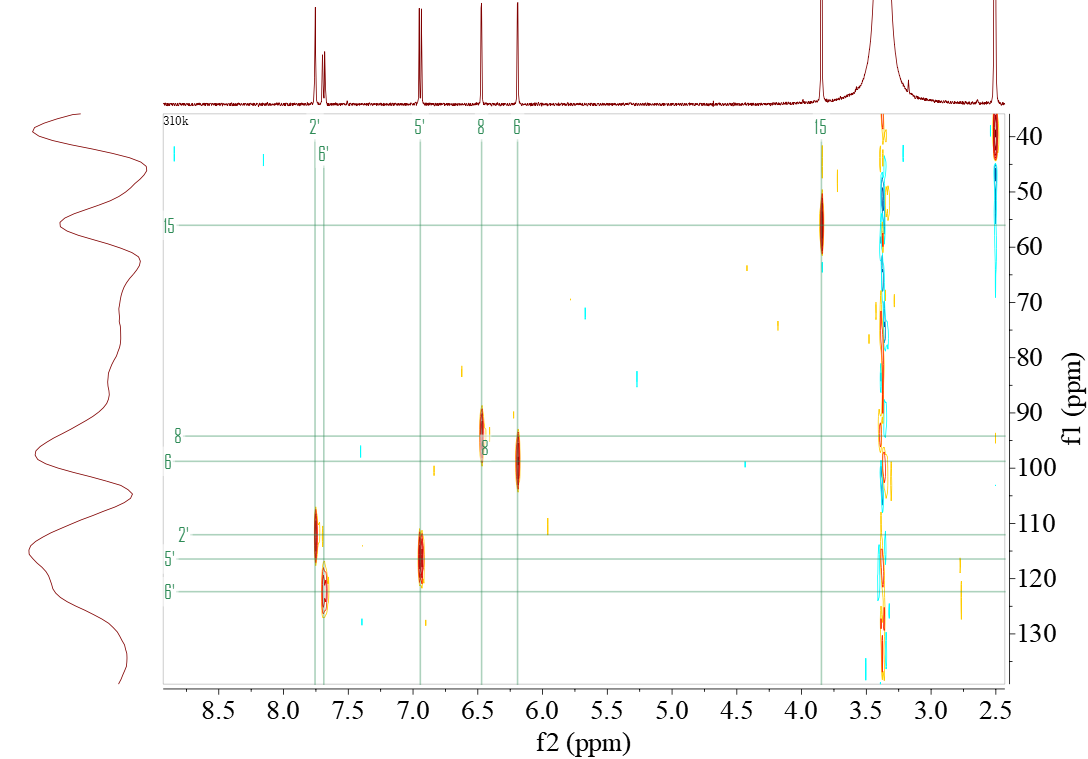


**Figure S7.** The HSQC (^13^C,125 MHz, DMSO-d_6_ ) spectrum of Compound III.


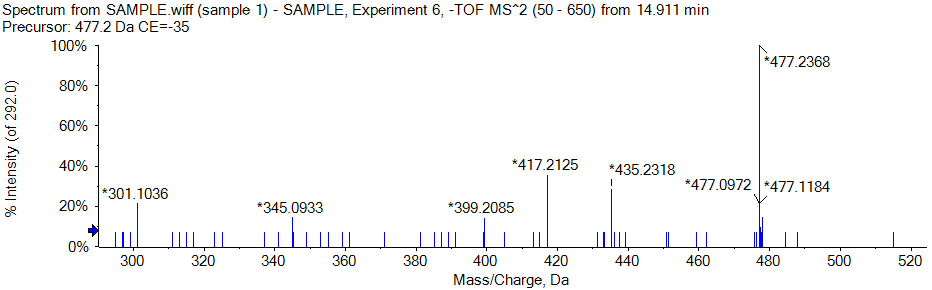


**Figure S8.** The GC-MS spectrum of Compound IV.


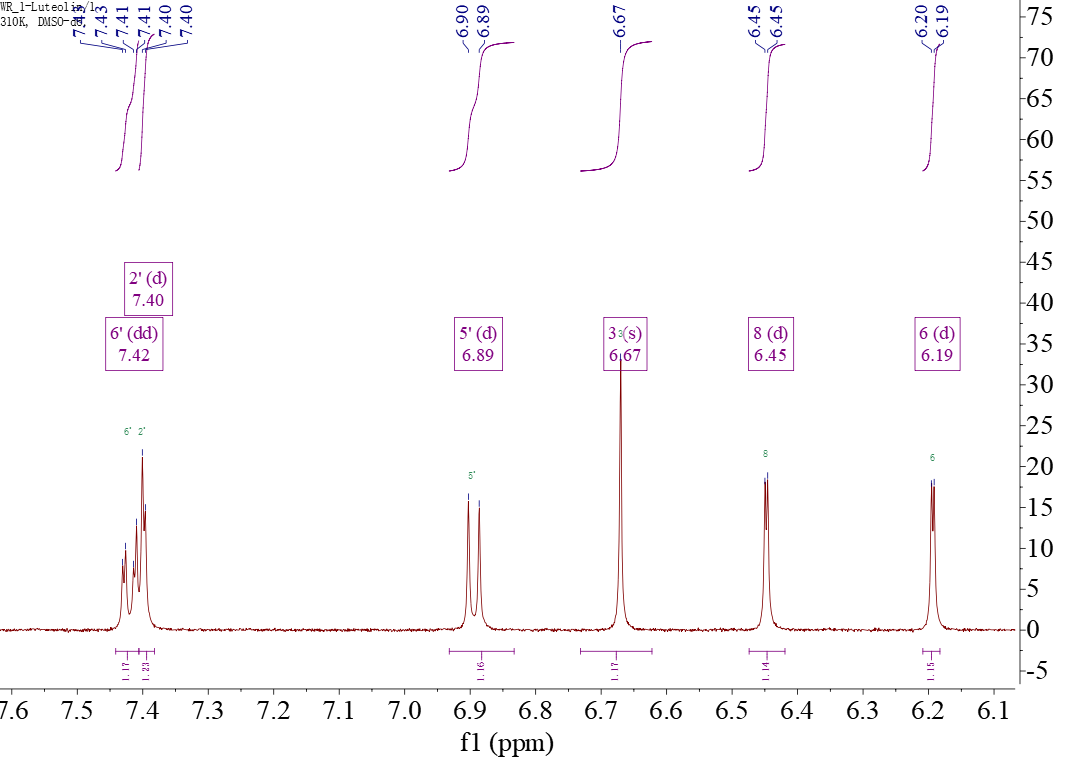


**Figure S9.** The ^1^H NMR spectrum (500 MHz, DMSO-d_6_) of Compound IV.


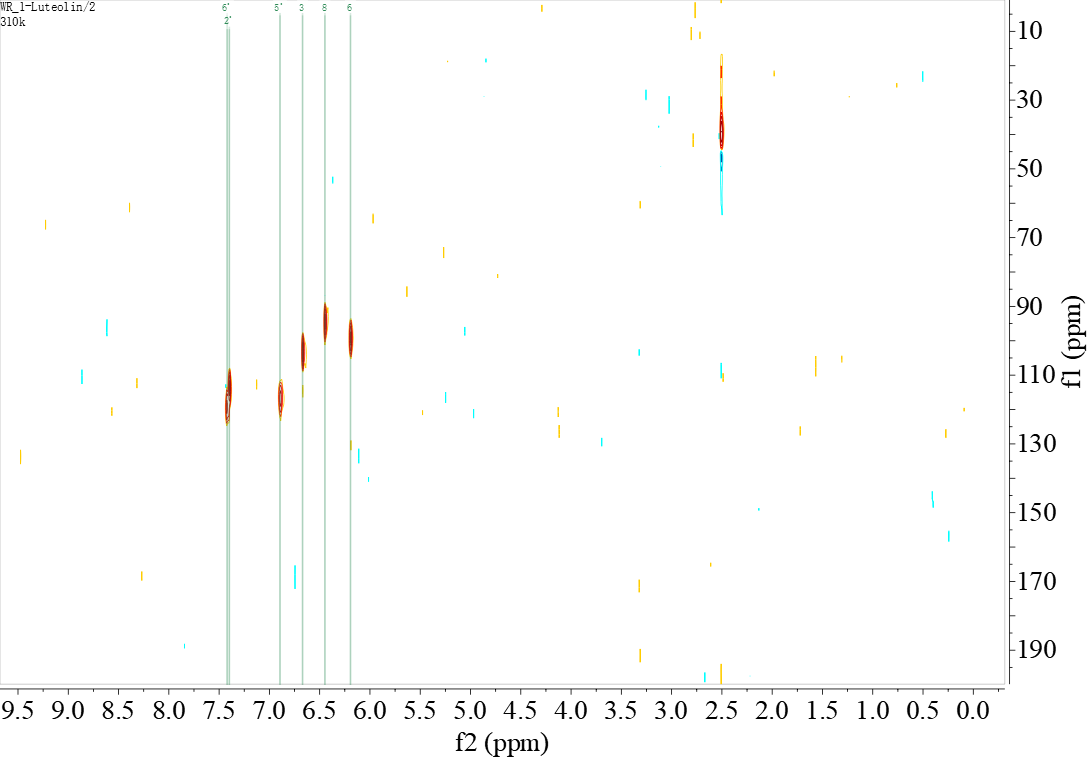


**Figure S10.** The HSQC (^13^C,125 MHz, DMSO-d_6_ ) spectrum of Compound IV.


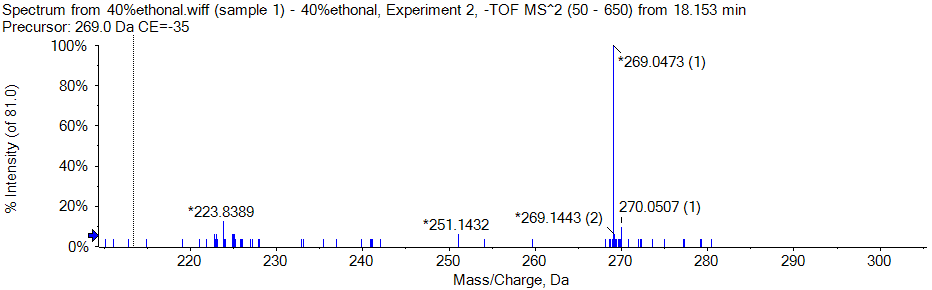


**Figure S11.** The GC-MS spectrum of Compound V.


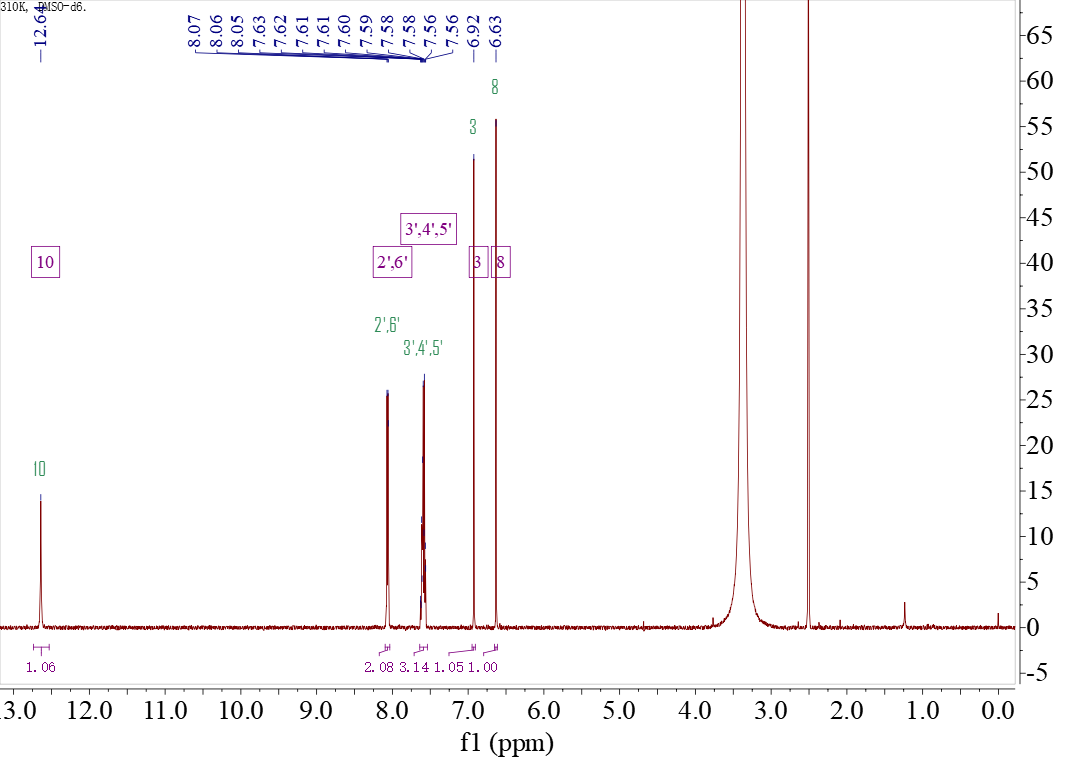


**Figure S11.** The ^1^H NMR spectrum (500 MHz, DMSO-d_6_) of Compound V.


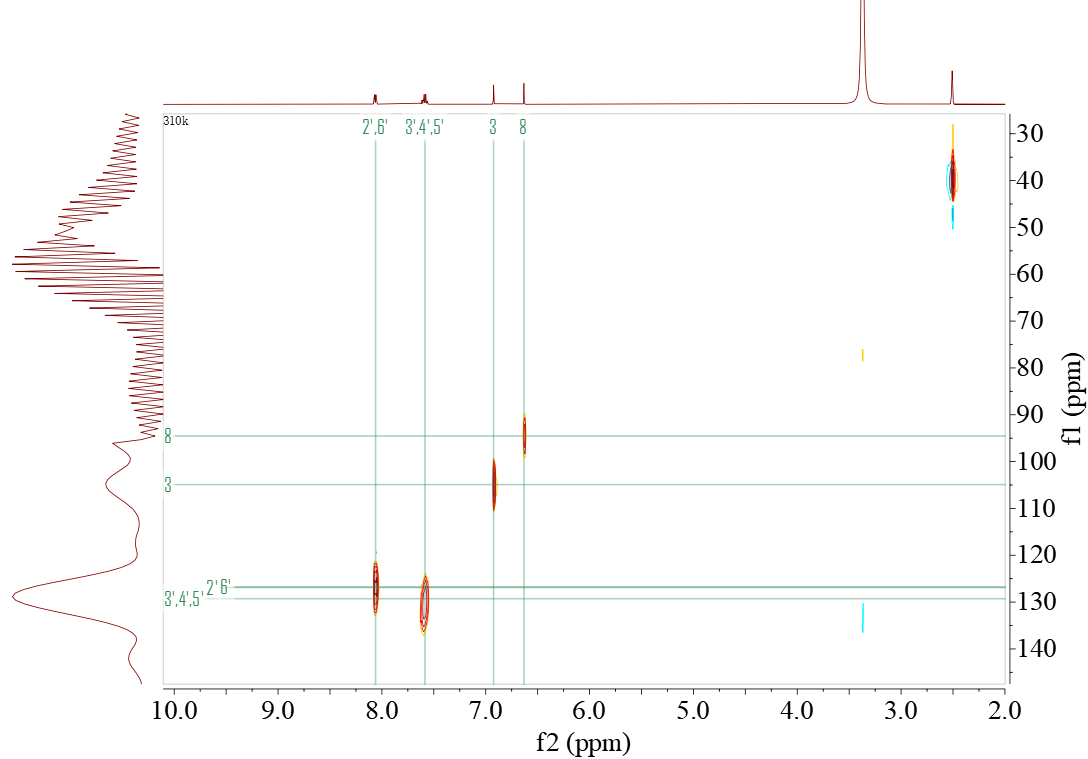


**Figure S12.** The HSQC (^13^C,125 MHz, DMSO-d_6_ ) spectrum of Compound V.


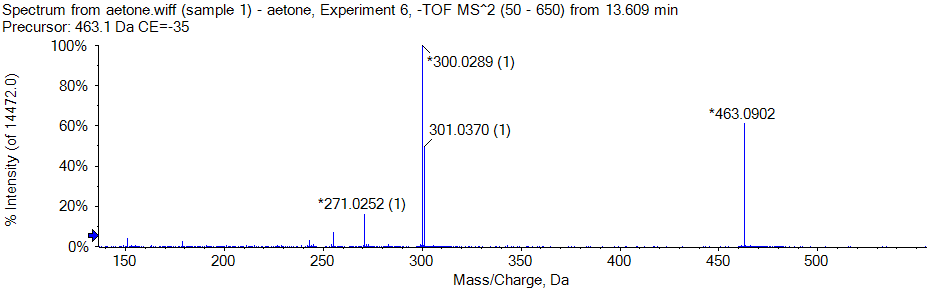


**Figure S13.** The GC-MS spectrum of the Compound VI.


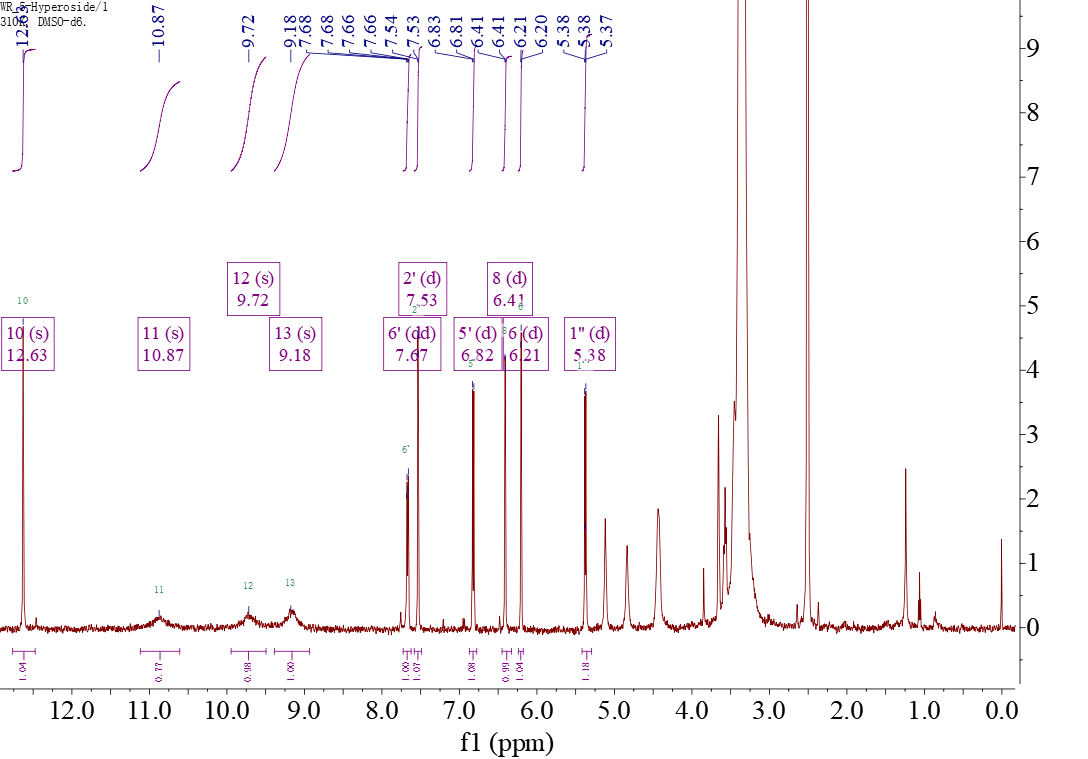


**Figure S14.** The ^1^H NMR spectrum (500 MHz, DMSO-d_6_) of Compound VI.
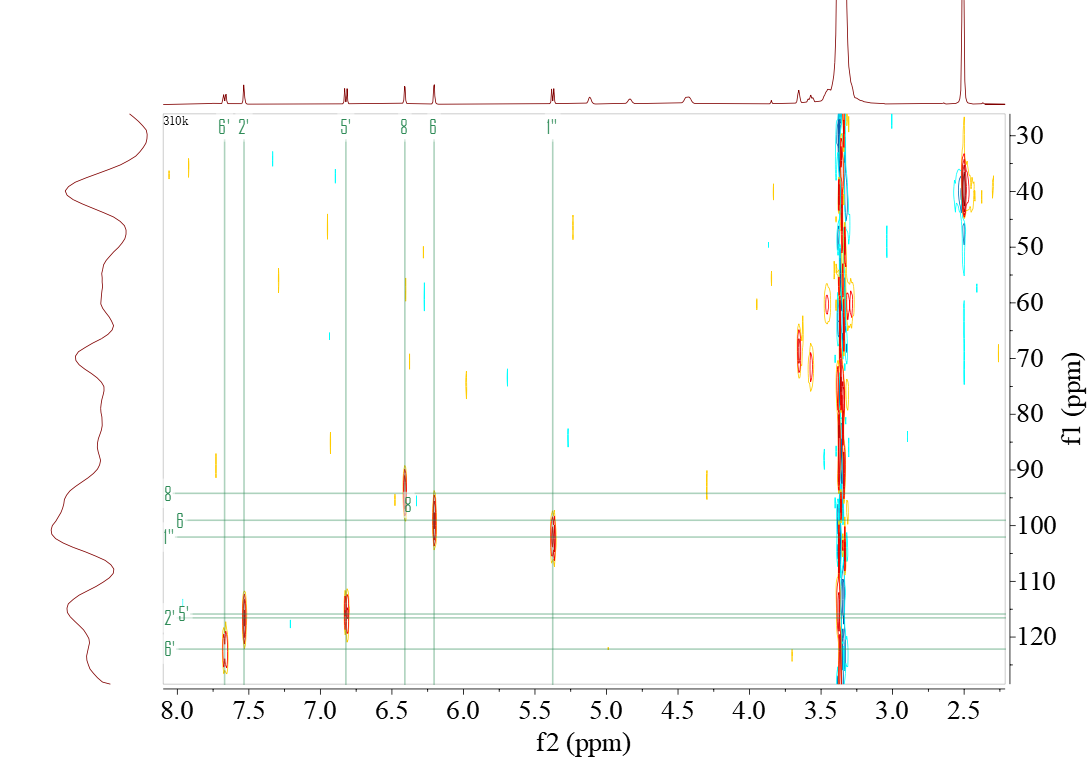


**Figure S15.** The HSQC (^13^C,125 MHz, DMSO-d_6_ ) spectrum of Compound VI.


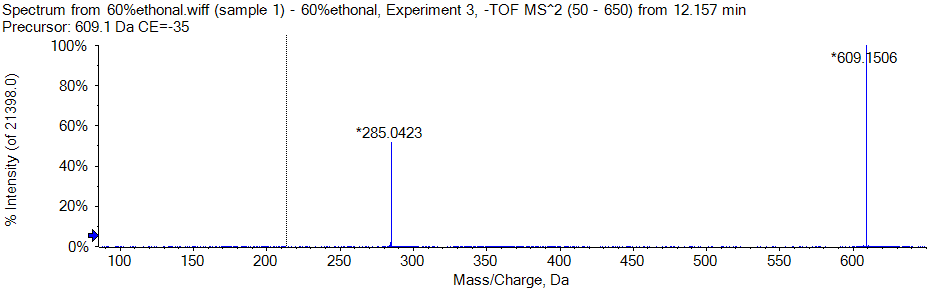


**Figure S16.** The GC-MS spectrum of Compound VII.


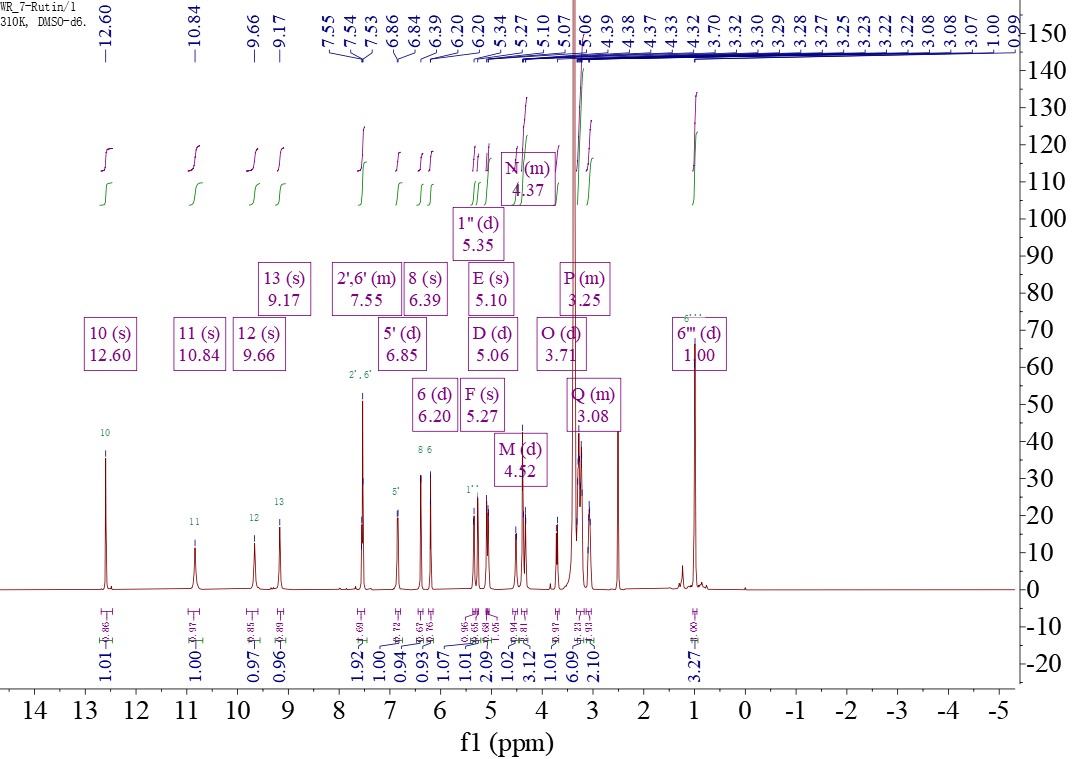


**Figure S17.** The ^1^H NMR spectrum (500 MHz, DMSO-d_6_) of Compound VII.


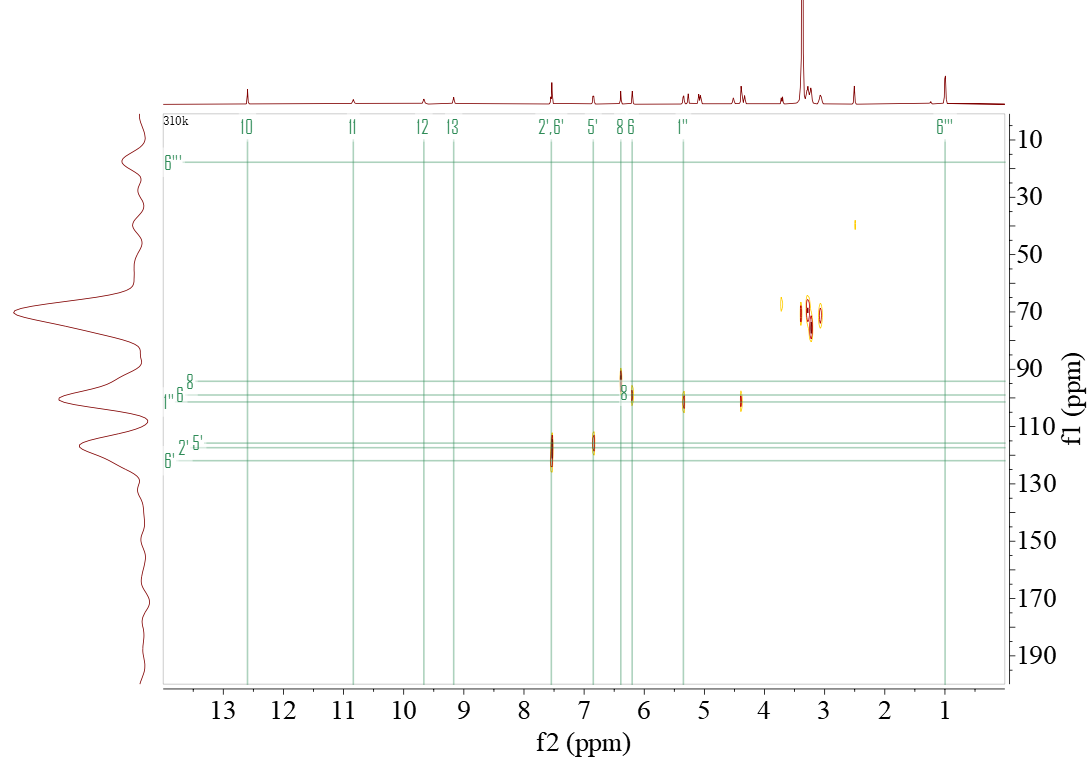


**Figure S18.** The HSQC (^13^C,125 MHz, DMSO-d_6_ ) spectrum of Compound VII.

**Assessment of antioxidant activity**

**Figure S19.** UV-vis absorption spectra for measuring the reaction between different flavonoid compounds and DPPH.

**Table S3.** Liner relationship between concentration of antioxidant and scavenging activity.

| Sample | Linear Equation | R^2^ | IC_50_ |
| --- | --- | --- | --- |
| Quercetin | Y = 5.9696 x + 1.8239 | 0.9973 | 8.07 |
| Hesperetin-5 '-O-β-rhamnoglucoside | Y = 5.7104 x + 0.1652 | 0.9945 | 8.72 |
| Hesperetin 7-glucuronide | Y = 3.5306 x + 2.3531 | 0.9957 | 13.49 |
| Baicalein | Y = 3.2095 x + 0.2277 | 0.9981 | 15.5 |
| Kaempferol-3-glucoside | y = 2.3246 x-1.3727 | 0.9973 | 22.1 |
| Hyperoside | y = 1.6876 x-2.9867 | 0.9894 | 31.39 |
| Rutin | y = 1.5872 x-0.0651 | 0.9866 | 31.54 |


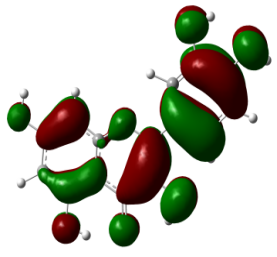

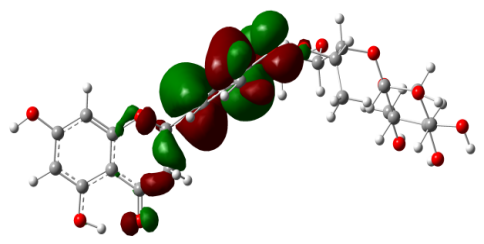

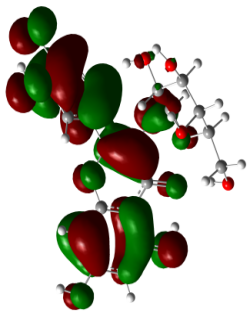


**(a)**

**(b)**

**(c)**

1. **Quercetin-3’OH (b) Hesperetin-5'-O-β-rhamnoglucoside-3’CH_3_ (c) Hesperetin-7-glucuronide-3’OCH_3_**

**Figure S20.** HOMO distribution map of frontier orbital of three flavonoids (red and green colors in the map respectively represent the corresponding phases when two positive and negative charges in molecular orbital are different).

**References**

1. Hye, J.H., Ji, S.Lee., Sun, A.P., Jun, B.A., Hyeon, G. L., Extraction optimization

and nanoencapsulation of jujube pulp and seed for enhancing antioxidant activity. *Colloids and Surfaces B: Biointerfaces* **130**,93-100, [https://doi.org/10.1016/j.col](https://doi.org/10.1016/j.colsurfb.2015.03.050)

[surfb.2015.03.050](https://doi.org/10.1016/j.colsurfb.2015.03.050) (2015).

1. Berkani, F., Dahmoune, F., Achat, S. et al. Response Surface Methodology

Optimization of Microwave-Assisted Polysaccharide Extraction from Algerian Jujube (Zizyphus lotus L.) Pulp and Peel. *J Pharm Innov* **16**, 630-642, [https://doi.](https://doi.org)

[org](https://doi.org)/10.1007/s12247-020-09475-9 (2021).

1. Kassama, L.S., Shi, J., Mittal, G.S. et al. Optimization of supercritical fluid

extraction of lycopene from tomato skin with central composite rotatable design

model. *Separation＆ Purification Technology* **60**, 278-284, [https://doi.org/1](https://doi.org/10.1016/j.seppur.2007.09.005)

[0.1016/j.seppur.2007.09.005](https://doi.org/10.1016/j.seppur.2007.09.005) (2007).

1. Cai, C., Ma, J., Han, C., Jin, Y., Zhao, G. et al.. Extraction and antioxidant

activity of total triterpenoids in the mycelium of a medicinal fungus, Sanghuangporus sanghuang. *Sci Rep* **15**, 7418, https://doi. 10.1038/s41598-019-

43886-0 (2018).

5. Yi, G.Z., Huan, K., Sheng, X. C., Kiran, T., Shao, y. et al. Comparison of phenolic compounds extracted from Diaphragma juglandis fructus, walnut pellicle, and flowers of Juglans regia using methanol, ultrasonic wave, and enzyme assisted-extraction. *Food Chemistry* **321**,126672, https://doi.org/10.1016/

j.foodchem.2020.126672 (2020).

6. Cheng, L., Yang, Q., Chen, Z., Zhang, J., Chen, Q.et al. Distinct Changes of Metabolic Profile and Sensory Quality during Qingzhuan Tea Processing Revealed by LC-MS-Based Metabolomics. *J Agric Food Chem* **68**, 4955-4965, https://doi,10.1021/acs.jafc.0c00581 (2020).

7. Pereira, C.G., Clifford, M.N., Polyviou,T., Ludwig, I.A., Alfheeaid, H.et al.

Plasma pharmacokinetics of (poly)phenol metabolites and catabolites after

ingestion of orange juice by endurance trained men. *Free Radic Biol Med* **160**, 784-795, https://doi,10.1016/j.freeradbiomed.2020.09.007(2020).

8. Yue,T., Chen, R., Chen, D., Liu, J. et al. Enzymatic Synthesis of Bioactive

O-Glucuronides Using Plant Glucuronosyltransferases. *J Agric Food Chem* **67**,

6275-6284. https://doi,10.1021/acs.jafc.9b01769 (2019).

9. Alvarez-Fernandez, M.A., Hornedo-Ortega, R., Cerezo, A.B., Troncoso, A.M. &

Garcia-Parrilla, M.C. Effects of the strawberry (Fragaria ananassa) puree

elaboration process on non-anthocyanin phenolic composition and antioxidant

activity. *Food Chem* **164**, 104-112, https://doi,10.1016/j.food chem.2014.04.116

(2014).

10. Didem, Ş., Suat, S., Michal, Š., Burak, B,.Tibor, L.et al. Potential of Potentilla inclinata and its polyphenolic compounds in α-glucosidase inhibition: Kinetics and interaction mechanism merged with docking simulations, *Inter J Bio Macro*

**108**, 81-87, https://doi.org/10.1016/j.ijbiomac.2017.11.151(2018).

11. Vedpal, J.U., Wadhwani, A. & Dhanabal, S.P. Isolation and characterization of flavonoids from the roots of medicinal plant Tadehagi triquetrum (L.) H.Ohashi. *Nat Prod Res* **34**, 1913-1918, https://doi,10.1080/14786419.2018.1561679 (2018).

12. Li, J., Han, L. & Chao, J. Preparation and characterization of the inclusion

complex of baicalein with γ-cyclodextrin: an antioxidant ability study. *J Inclus*

*Phen Macr Chem* **73**, 247-254, https://doi,10.1007/s10847-011-0048-x (2011).

13. Karakaya, S., Süntar, I., Yakinci, O.F., Sytar, O. & Guvenalp, Z. In vivo

bioactivity assessment on Epilobium species: A particular focus on Epilobium

angustifolium and its components on enzymes connected with the healing

process. *J Ethnopharmacol* **262**, 113207, https://doi,10.1016/j.jep.2020.113207

(2020).

14. Lee, J., Jang, D.S., Yoo, N.H., Lee, Y.M. & Kim, J.S. Single-step separation of

bioactive flavonol glucosides from Osteomeles schwerinae by high-speed

counter-current chromatography. *J Sep Sci* **33**, 582-6, https://doi,10.1002/jssc.

200900693 (2010).

15. Joshua, M.H., Asim, M., Jonathan, F., Ammar, S.A. & Kimberly, L.C.Quantifi-

cation of Chlorogenic Acid and Hyperoside Directly from Crude Blueberry

(Vaccinium angustifolium) Leaf Extract by NMR Spectroscopy Analysis: Sing-

le-Laboratory Validation, *Journal of AOAC INTERNATIONAL* **95**, 1406-1411,

https://doi.org/10.5740/jaoacint.11-415 (2012).

16. Zhang, W., Xu, M., Yu, C., Zhang, G. & Tang, X. Simultaneous determination of vitexin-4''-O-glucoside, vitexin-2''-O-rhamnoside, rutin and vitexin from hawthorn leaves flavonoids in rat plasma by UPLC-ESI-MS/MS. *J Chromatogr B Analyt Technol Biomed Life Sci* **878**, 1837-1844, https://doi,10.

1016/j.jchromb.2010.05.023 (2010).

17. Yang, J., Qian, D., Jiang, S., Shang, E.X. & Duan, J.A. Identification of rutin deglycosylated metabolites produced by human intestinal bacteria using UPLC-Q-TOF/MS. *J Chromatogr B Analyt Technol Biomed Life Sci*, **898**, 95-100, https://doi,10.1016/j.jchromb.2012.04.024 (2012).
